# Supplementary material for: Automatic wavelet-based 3D nuclei segmentation and analysis for multicellular embryo quantification
Source: Sci Rep. 2021 May 10;11:9847. doi: 10.1038/s41598-021-88966-2 (PMC8110989; doi:10.1038/s41598-021-88966-2)
Supplement: Supplementary file 1 — Supplementary Information 1. [file 41598_2021_88966_MOESM1_ESM.pdf]

The ARRIVE Essential 10: Compliance Questionnaire

Use this questionnaire to evaluate how well a manuscript complies with the ARRIVE Essential 10. It can be applied to any manuscript describing comparative experiments in living animals, by assessors such as journal staff, editors, or peer reviewers.

Item	Question(s)	Answers
1 Study Design	Are all experimental and control groups clearly identified?	☒ Yes, for at least one experiment<br>☐ No
	Is the experimental unit (e.g. an animal, litter or cage of animals) clearly identified?	☒ Yes, for at least one experiment<br>☐ No
2 Sample Size	Is the exact number of experimental units in each group at the start of the study provided (e.g. in the format '*n*=')?	☒ Yes, for at least one experiment<br>☐ No
	Is the method by which the sample size was chosen explained?	☒ Yes, for at least one experiment<br>☐ No
3 Inclusion & Exclusion Criteria	Are the criteria used for including and excluding animals, experimental units, or data points provided?	☒ Yes, for at least one experiment<br>☐ No
	Are any exclusions of animals, experimental units, or data points reported, or is there a statement indicating that there were no exclusions?	☒ Yes, for at least one analysis<br>☐ No
4 Randomisation	Is the method by which experimental units were allocated to control and treatment groups described?	☒ Yes, for at least one experiment<br>☐ No
5 Blinding	Is it clear whether researchers were aware of, or blinded to, the group allocation at any stage of the experiment or data analysis?	☒ Yes, for at least one experiment<br>☐ No
6 Outcome Measures	For all experimental outcomes presented, are details provided of exactly what parameter was measured?	☒ Yes, for at least one experiment<br>☐ No
7 Statistical Methods	Is the statistical approach used to analyse each outcome detailed?	☒ Yes, for at least one analysis<br>☐ No
	Is there a description of any methods used to assess whether data met statistical assumptions?	☒ Yes, for at least one analysis<br>☐ No<br>☐ Not applicable
8 Experimental Animals	Are all species of animal used specified?	☒ Yes, for at least one experiment<br>☐ No
	Is the sex of the animals specified?	☐ Yes, for at least one experiment<br>☐ No<br>☒ Not applicable to species
	Is at least one of age, weight or developmental stage of the animals specified?	☒ Yes, for at least one experiment<br>☐ No
9 Experimental Procedures	Are both the timing and frequency with which procedures took place specified?	☒ Yes, for at least one experiment<br>☐ No
	Are details of acclimatisation periods to experimental locations provided?	☒ Yes, for at least one experiment<br>☐ No
10 Results	Are descriptive statistics for each experimental group provided, with a measure of variability (e.g. mean and SD, or median and range)?	☒ Yes, for at least one experiment<br>☐ No<br>☐ Not applicable to the type of data collected
	Is the effect size and confidence interval provided?	☒ Yes, for at least one experiment<br>☐ No<br>☐ Not applicable to the type of analysis used

Notes on questionnaire design

The ARRIVE guidelines are a useful resource for authors preparing manuscripts describing animal research, and also provide a framework to evaluate the transparency of those manuscripts. To assess reporting quality, numerous studies have in the past sought to operationalise reporting guidelines (including ARRIVE). Typically, this involves scoring a manuscript's degree of compliance with guideline items in a binary fashion (e.g. an item is either not reported or reported) [1-3], a graded fashion (e.g. not, partially, or completely reported) [4,5], or a combination of the two [6].

This questionnaire has been designed to be as concise and user-friendly as possible. The number of questions used to assess a manuscript's compliance has been kept to a minimum, and in most cases each question is designed to be answered in a binary fashion. Compliance with some Essential 10 sub-items is inherently impossible to judge in this way, instead requiring a subjective judgement on the level of detail provided. For this reason, not all sub-items are represented by a question in this questionnaire.

To facilitate binary answers, it has been necessary to identify the minimum information in a manuscript sufficient to comply with each question. The strengths of this approach include the relatively short length of the questionnaire (and the correspondingly low time burden of using it), and the avoidance of ambiguity that would arise from a graded answering system, in which an intermediate score (e.g. 'partially/insufficiently reported') could denote a number of distinct deficiencies in compliance with an item (e.g. either only part of the item was complied with, or only the reporting of some experiments in the manuscript complied with the item.)

Limitations of this approach centre on the necessity to identify the minimum information sufficient to comply with each question. In some cases, this has resulted in questions that require a guideline sub-item's criteria to have been fulfilled in the reporting of only one experiment in a manuscript. As a result, not all experiments in a manuscript may be described in a way that fulfils that criterion, despite the manuscript being considered to comply with the guidelines overall.

References

1. Hair *et al* (2020). *Res Integ Peer Rev*. doi: 10.1186/s41073-019-0069-3
2. Tihanyi *et al* (2019). *J Surg Res*. doi: 10.1016/j.jss.2018.10.038
3. Zhao *et al* (2020). *BMC Vet Res*. doi: 10.1186/s12917-020-02664-1
4. Han *et al* (2017). *Plos One*. doi: 10.1371/journal.pone.0183591
5. Chatzimanouil *et al* (2019). *J Am Soc Nephrol*. doi: 10.1681/ASN.2018050515
6. Leung *et al* (2018). *Plos One*. doi: 10.1371/journal.pone.0197882

Fig. S1. Illustration of wavelet-based segmentation method in an optical section of a whole-mount zebrafish embryo. (A, A1-3) Whole-mount embryo z-slice nuclear DAPI-staining raw images of germ ring-stage (5.7 hpf) zebrafish and three red-boxed regions. Image data were acquired with a Zeiss LSM 800 confocal microscope with 20X water lens. (B, B1-3) Wavelet coefficient matrixes after applying 2D CWT on raw images in (A). The blue color corresponds to the negative coefficient value region, and the yellow color corresponds to the positive coefficient value region. 2D Mexican hat function was selected as the mother wavelet function. (C, C1-3) 2D segmentation masks (yellow regions) were obtained after applying multi-scale object identification on wavelet coefficient matrixes in (B).

Fig. S2. Limitations for nuclei segmentation on whole embryo confocal imaging. (A, B) Nuclei with clear boundaries high contrast and low contrast and non-uniform background intensity nuclei. (C) Whole zebrafish embryo z-slice nuclear DAPI staining with confocal Intensity attenuation effect, and the nuclei segmentation results using wavelet-based segmentation method. Green boundaries show nuclei segmentation masks and nuclei with inhomogeneous intensity can be detected.

Fig. S3. Nuclei segmentation and RNA segmentation results using wavelet-based segmentation method. (A) C.elegans developing embryo nuclear DAPI staining by light-sheet microscope with segmentation masks marked by green boundaries (B) Pancreatic Stem Cells on a Polystyrene substrate, (C) GFP-GOWT1 mouse stem cells, and (D)(E) Whole mount zebrafish embryo z-slice bmp2b mRNA expression at 5.7hpf, and bmp RNA spot segmentation results using wavelet-based segmentation method in different z planes(left and right). Insets: enlarged views of two while box regions. (B,C) data from the Cell Tracking Challenge http://celltrackingchallenge.net/2d-datasets/

Fig. S4. Workflow of multi-scale object identification step in wavelet-based segmentation method. (A) Zebrafish embryo nuclear DAPI staining raw images (5.7hpf). We apply 2D continuous wavelet transform (2D CWT) on this raw image with three wavelet scale factors s1, s2, s3. (B) Three wavelet coefficient maps with wavelet scale factors s1, s2, s3. Contour color represents intensity of the corresponding wavelet coefficient. The green intensive

contoured ridges region in three wavelet coefficient maps are almost identified and can be correlated with nuclei region in raw image. (C) Local minimums were determined on three wavelet coefficients map after the H-minima transform. If local minimums appeared in the same positions on all wavelet coefficient maps, they were identified as potential nuclei peak locations and the corresponding zero-value cross sections are calculated. (D) In this step we calculated the 2D masks intersection over Union (IOU) of all zero-value cross sections from all wavelet coefficient maps. If the difference in cross section areas is less than 10%, they averaged and were identified as nuclei 2D masks. (E) The final segmentation mask after multi-scale object identification step.

Fig. S5. First division step based on wavelet coefficient center positions on z-slice of nuclei 3D blob. (A) Five z-slice wavelet coefficient maps of segmented nuclei 3D blob from top (z = 7) to bottom (z = -1). Color represents intensity of the corresponding wavelet coefficient from positive value (yellow) to negative value (deep blue). Red cross marks the center position (local minimum position) on each wavelet coefficient map z-slice in 3D blob. Here the z value came from z axis of whole embryo before 3D rotation. (B) If there are more than one center positions found or the shift of the center positions between neighboring Z slices is bigger than nuclei radius, we assigned a new nuclei object by cutting the median line between two centers, or assigned new nuclei in the case of center displacement. In (A) we found two center position shift between z=5 to z=3 and z=1 to z=-1, and marked using red arrow. So we divided the original 3D nuclei blob (left) into three nuclei 3D blobs (right) with three new nuclei centers (red dot) and displayed using different color. (C) Three examples show the 3D nuclei blobs after the first division step. Red box on the third 3D nuclei blob (right) shows the nuclei overlapping in z direction cannot be divided in the first division step.

Fig. S6. Second division step in z direction and the summary of blob size distribution before and after division steps. (A) The 3D nuclei blob (left) shows the nuclei overlapping in z direction which didn't divide appropriately in the first division step because there is no center position shift between neighboring Z slices. In the second division step, wavelet coefficient yz and xz plane are examined for center positions for every 3D nuclei. If two center positions are found on both yz and xz planes (center left and center right), original 3D nuclei blob will be

divided into two new blobs from the middle plane of two center positions (right). (B) Remove the 3D nuclei blobs smaller than nuclei size × 0.2. (C) Blob size distributions in three axis directions (upper: x-direction, center: y-direction, lower: z-direction) before the first division step (left column), and after the first division step (center column), and after the second division step (right column) for all nuclei segmented using wavelet-based segmentation method in whole embryo. The final size distributions in three directions are all similar to normal distributions (right column).

Fig. S7. The workflow of synthetic data generator and process to add noise in WaveletSEG. The synthetic image generator in WaveletSEG can generate synthetic images or synthesis overlapping images by providing image number, image size, synthetic nuclei number, nuclei radius and intensity and their randomness. For overlapping images, we iteratively create pairing nuclei with distances between two nuclei are smaller than nuclei radius × 1.5. After initial synthesis image dataset are created, Gaussian noise, salt and pepper noise with different noise level can be added into images, or apply down-sampling or up-sampling to those images.

Fig. S8. Ground truth image dataset we used and the ground truth labeling GUI and 2D segmentation viewer. (A) Five 3D ground truth of 400x400x24 voxel region nuclear DAPI staining raw images from 4.7hpf Zebrafish embryo (first three images), and raw images 400x400x24 voxel region of 4.7hpf Zebrafish embryo in pSMAD datasets. (B) To build the ground truth dataset using our own images to validate the segmentation method, we introduced Raw_image sub-GUI, a 3D nuclei annotation tool which provides a convenient and efficient way to label 3D nuclei, and can also be used to evaluate nuclei segmentation results. The left window of the Raw_image shows the raw image with side bar to view different z-slice. (C) The right window shows both raw image and segmentation labels, and provides a very convenient way to label 3D nuclei. When user clicking inside the right-side window, pop-up GUI will show up and display every z-slice in this position. User can select the nuclei center and do labeling.

Fig. S9. WaveletSEG main functions. WaveletSEG main function block consists of five main steps which can run independently including 1. Nuclei identification, 2. Embryo orientation, 3. Shape classification, 4. Profile extraction, and 5. Time lapse steps. In the nuclei identification step, whole embryo nuclei are segmented using wavelet-based segmentation method, and average nuclear fluorescent intensity is also calculated. User can do an intensity calibration by using nuclei nearby intensity. In IO system block, User can directly import microscope image files or intermediate data files into WaveletSEG, and save or output data results or figures directly from the GUI. In the embryo orientation step, an individual embryo is rotated in 3D automatically according to some specific chemical marker for the axis such as chd mRNA. Embryo coordinate value and 3D topology features for each nucleus is also calculated after rotation to provide positional information. To integrate multiple embryos, CPD registration is applied here. In shape classification step, we defined shape-based rules to classify nuclei into spherical nuclei, irregular shape nuclei, elliptical nuclei, and division nuclei to estimate cell proliferation and cell cycle phases. Unsupervised learning clustering such as SOM network is also included to cluster nuclei based on nuclei size and shape. In profile extraction step, we project the nuclei quantification and features into one averaged distributed sphere plane to enhance accuracy distribution. We also developed a set of segmentation validation tools in WaveletSEG including synthetic data generator, 3D ground truth labeling system, 2D and 3D segmentation viewer sub-GUI, and segmentation method comparison extension GUI. In WaveletSEG data visualization block, 3D segmentation and quantification or other results such as embryo topology features can be easily displayed or saved in WaveletSEG data visualization system. Users can also create scatter plots by selecting menu options to explore the relationship between them.

Fig. S10. Raw image3D sub-GUI. Raw image3D is the 3D segmentation data visualization GUI to check and validate the segmentation results in WaveletSEG, and users can execute this sub-GUI by clicking the button “Raw3D images”. Blue points inside the main data visualization window (Right) are segmented nuclei positions are displayed as 3D point cloud, and horizontal plane is the raw image z-slice enable user to validate and compare 3D segmented nuclei with original raw image z-slice. Left side of this sub-GUI is the control panel to display regional nuclei or change into different images or channels.

Fig. S11. Compare SEG sub-GUI. Compare SEG is designed to validate the segmentation results or compare segmentation results using different segmentation methods in WaveletSEG. User can execute this sub-GUI by clicking the button “Compare SEG” in WaveletSEG. Right side of this sub-GUI is the main data visualization window and segmented nuclei positions are displayed as 3D point cloud.

Fig. S12. Zebrafish embryo coordinate system in WaveletSEG. we developed the zebrafish embryo coordinate system including embryonic axes such as DV axis, AP axis, and DV center axis, Height axis, Height level axis, Depth level axis. If time-lapse images are available, there is an additional axis: time stage. The first five coordinates are values between zero to one, Depth level is the layer number counting from the outer shell, and the Time stage started from 1. DV center axis is defined by connecting ventral point to dorsal point through the top of embryo, angle is calculatedF and DV center value is from 0 to 1. Unlike the Height level axis which described the vertical distance from top to button of Embryo, we defined Height axis through the line connecting ventral point to dorsal point through the top of embryo to describe the embryo developmental process relative to which nuclei will grow from the top of the embryo to the embryo bottom.

Fig. S13. Data workflow from 3D whole embryo raw image to projected average intensity distribution in WaveletSEG. Step1: Import 3D raw image with both DAPI stained channel and pSMAD channel. Step2: Segmented nuclei point cloud by using wavelet-based segmentation method. Step3: Automatically rotation of embryo by using chd or pSMAD channel embryonic spatial distribution. Step3a (optional): Apply intensity calibration by using nearby nuclei intensity or do 3D rotation to adjust the embryo DV and AP axis in the right direction after wavelet-based segmentation. Step3b (optional): To merge embryos in the same stage into a representative embryo one by one using CPD. Step4: After 3D rotation and embryo center point is moved to position zero. Step5: Automatically remove the outermost layer and the innermost layer from embryo using depth level information. Step5a (optional): data analysis and data visualization in specific sub-region of whole embryo. Step6: We generated a hemispherical

surface with averaged distributed reference points using regular placement method, and projected each nucleus into this 2D hemispherical surface (projection surface). Step7: We projected the nuclei into reference points with the closest spherical coordination system angle (θ, ϕ) and created a projected average intensity and 3D topology.

Fig. S14. 3D topology features used in WaveletSEG. Eight 3D topology features are defined in WaveletSEG software to quantitatively describe topology changing through embryo developing process. Nuclei size is the total pixel number for every 3D segmented nuclei. We used four indexes to describe the spatial nuclei density distribution for the embryo, including density 3D, density 2D, neighbor 3D and neighbor 2D distance. Density is the total nuclei number with distance smaller than 30 pixels around one nucleus, and Neighbor distance is the average distance of the nuclei to the closest two nuclei. The difference between 3D and 2D is that we only calculate density in the same layer (depth level) for density 2D and neighbor 2D distance. Thickness is defined as the vertical distance from the innermost layer of embryo. The outer epithelial monolayer of a zebrafish embryo is called the enveloping layer (EVL). Here we defined the inner EVL and outlier EVL as the layer with depth level equal to one or the maximum values. H_sorting is the index to describe the dynamic process of nuclei movement by dividing every 500 nuclei from the top of the embryo to the embryo bottom.

Fig. S15. Cell cycle phase patterns based on nuclei shape classification results. (A) Examples of cell cycle phase in mitosis based on nuclei shape classification results, and the defined shape-based rules for cell cycle phase. (B) Nuclei count ratios for prophase, prometaphase, metaphase, and anaphase nuclei along the embryo height level in 4.7, 5.7, and 6.5 embryo developmental time. (C) Nuclei count ratios for prophase, prometaphase, metaphase, and anaphase nuclei along the embryo depth level in 4.7, 5.7, and 6.5 embryo developmental time. Green, red, and dark blue lines indicate mean value, and light green, light red, and light blue regions indicate one standard deviation region.

	WaveletSEG	DS	Point-wise	RACE	generic	MINS	ilastik	CellSegmentation3D	BioEmergences	CellSegm	MLS
Reference		38	41	36	51	33	62	63	64	65	66
Nuclei segmentation	●	●	●	●	●	●	●	●	●	●	●
Nuclei feature analysis	●	●		●	●						●
Time lapse/Tracking		●		●		●	●		●		
Graphical user interface	●			●		●	●				
Membrane SEG				●					●		
Synthetic dataset	●	●									
Quantification	●		●								
Nuclei shape analysis	●				●						
Cell fate/Cell lineage	●			●	●	●			●		●
Nuclei separation	●									●	●
Aberration calibration	●										
Data visualization	●					●		●	●		●
Count number	●		●			●					
Ground truth labeling	●										
Intensity calibration	●										
Cell registration	●										

Supplemental Table 1. Lists of nuclei segmentation embryonic imaging analysis tools.

Abbreviation	*Definition*
**ACMs**	active contour models
**AP**	anterior posterior axis
**BMP**	Bone Morphogenetic Protein
**CPD**	coherent point drift
**CWT**	continuous wavelet transform
**CT**	computerized tomography
**DAPI**	4′,6-diamidino-2-phenylindole
**DCL**	deep cell layer
**DV**	dorsal ventral axis
**DWT**	discrete wavelet transform
**DS**	derivatives sum method
**EVL**	the enveloping layer
**FN**	false negative
**FP**	false positive
**GMM**	gaussian mixture model
**GUI**	graphical user interface
**ND**	noise density
**PBS**	Phosphate-Buffered Saline
**PSMAD**	phosphorylated SMAD
**SNR**	signal-to-noise ratio
**TOTO**	in its entirety
**TP**	true Positive
**WTMM**	wavelet transform modulus maxima

Supplemental Table 2. Abbreviations list.

Algorithm Pseudo code for wavelet-based segmentation

Input: 3D image data

Output: 3D segmentation output

Load 3D image data

For all z planes do

    For s in wavelet scale range do

        wavelet coef map wt(s) <- 2D CWT for each Z plane of 3D image

        mask_wt(s) <- H-minima transform(wt(s))

    End for

    2D_Mask(z) = masks IOU({mask_wt(s)})

End for

3D_Mask’ <- 3D object alignment({2D_Mask(z)})

For all 3D segments do

    3D_Mask’’ <- First division(3D_Mask’)

    3D_Mask’’’ <- Second division(3D_Mask’’)

    3D segmentation output <- Delete outliers(3D_Mask’’’)

End for

Supplemental Table 3. Pseudo code for wavelet-based segmentation algorithm.

			Running time(sec)			
Testing Image	Image size(n)	File size	**Point-wise**	**Otsu's**	**DS**	**Wavelet**
Image1	504x508x117	88 MB	29.05±4.12	23.71±3.15	69.65±8.03.	66.65±6.81
Image2	502x503x199	147 MB	49.27±10.01	68.36±14.34	189.54±41.42	205.57±52.14
Image3	2048x2048x78	959 MB	341.96±69.58	391.24±73.05	729.25±95.12	794.06±84.37
Image4	1893x1960x120	1305 MB	431.78±89.02	554.12±94.51	1321.25±151.24	1415.47±131.12

Supplemental Table 4. Running time of four segmentation algorithms including point-wise, Otsu's, DS and wavelet methods on 3D imagings.

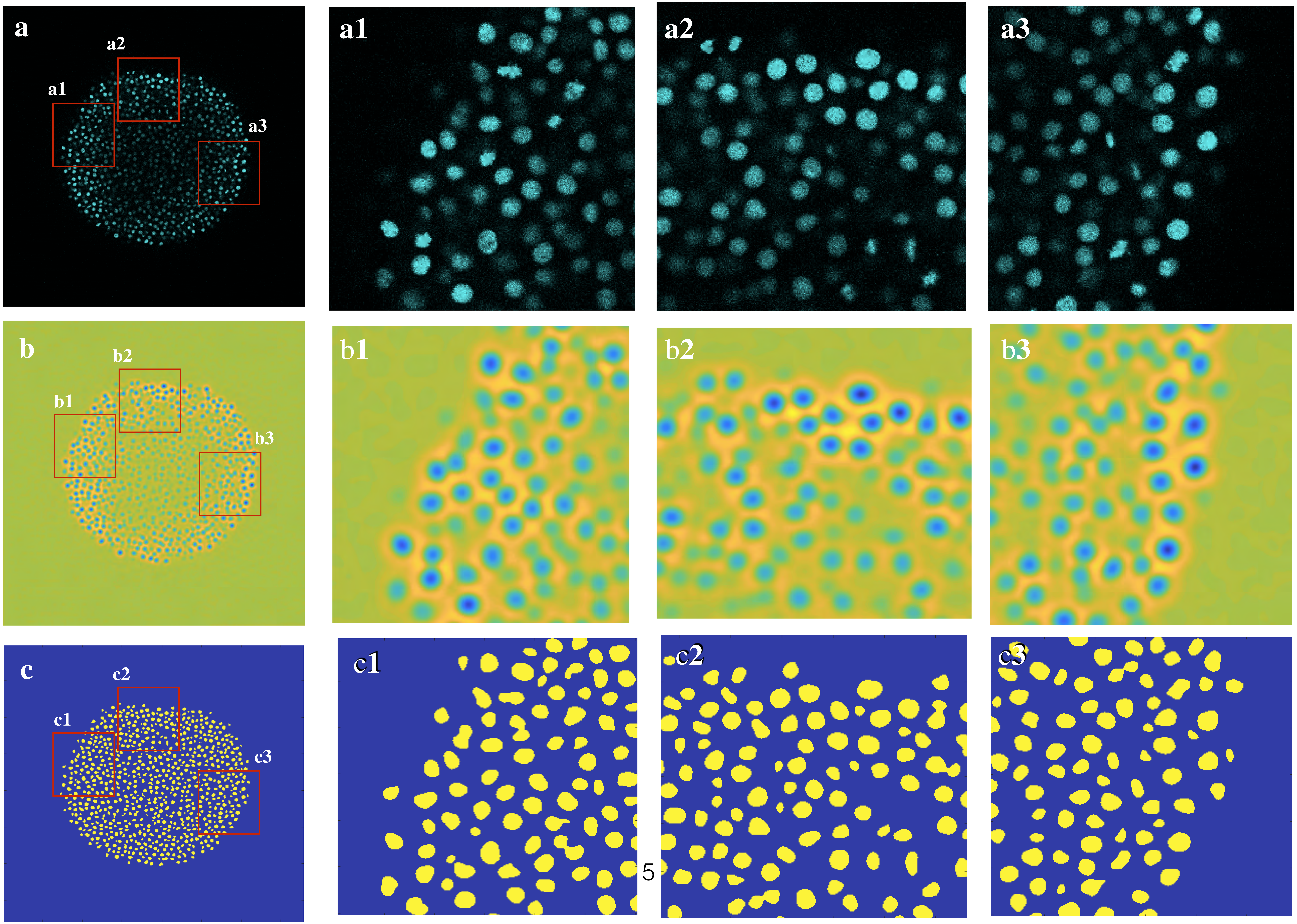

Supplemental Figure 1. Illustration of wavelet-based segmentation method in an optical section of a whole-mount zebrafish embryo.

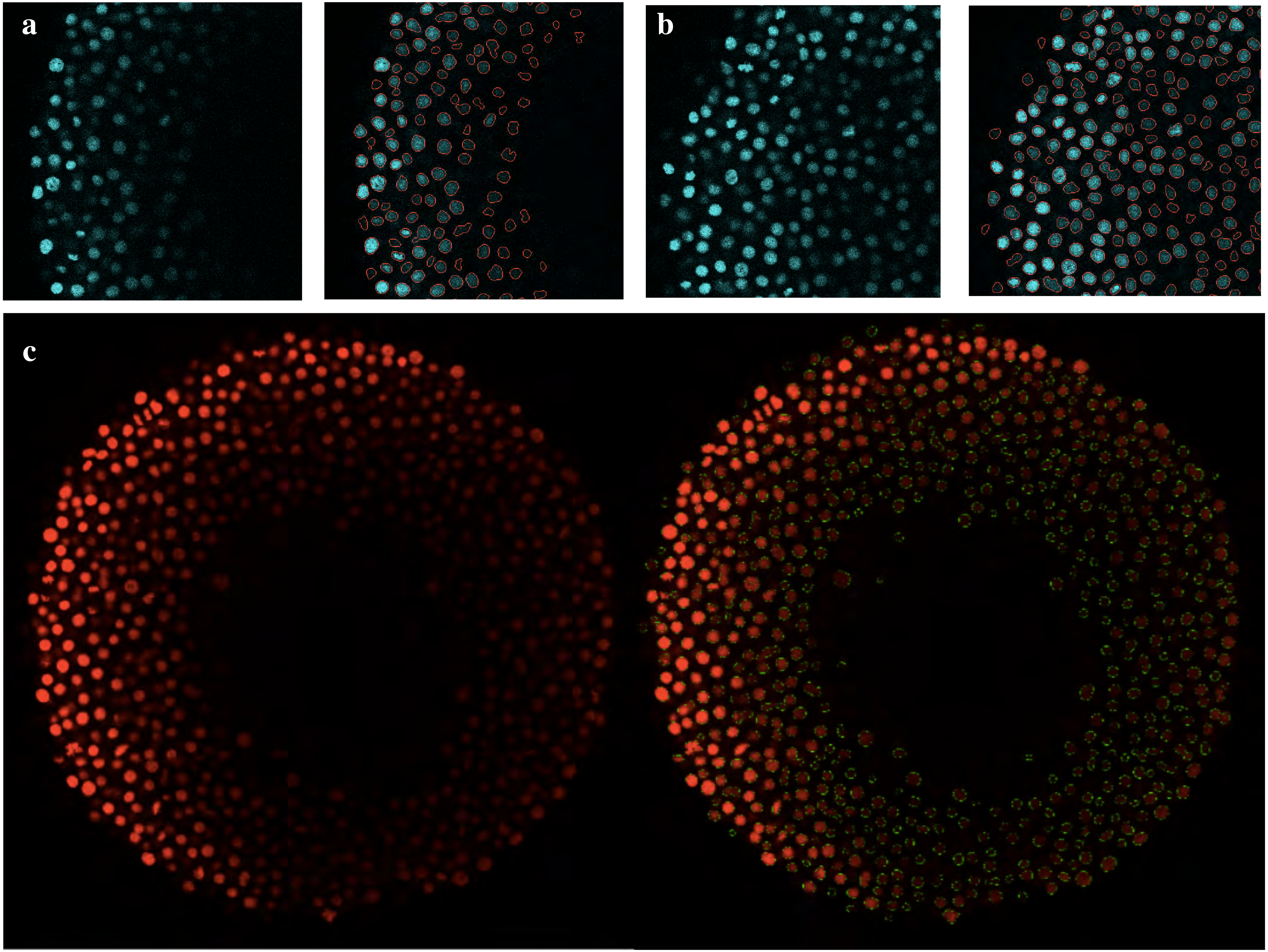

Supplemental Figure 2. Limitations for nuclei segmentation on whole embryo confocal imaging.

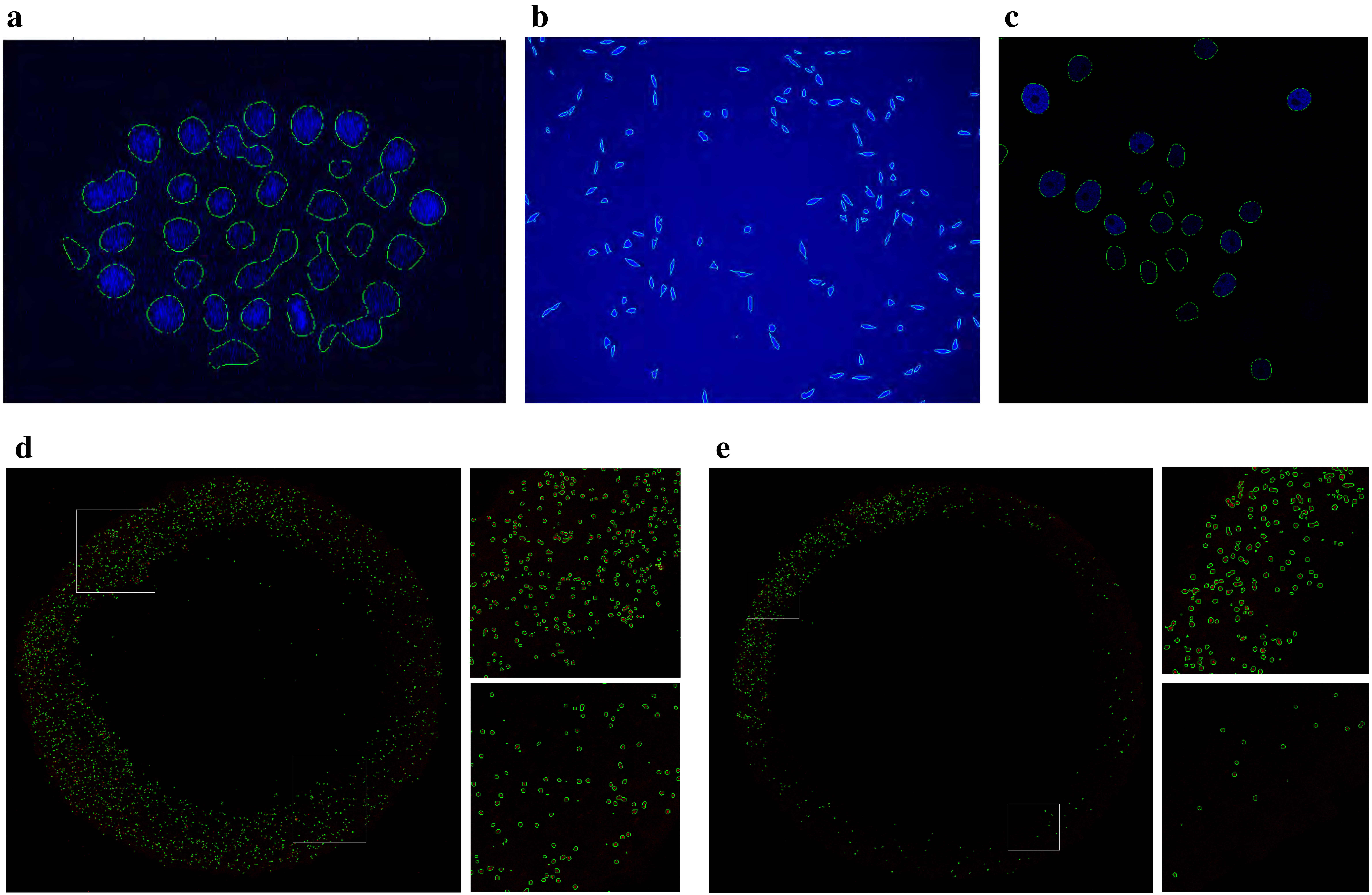

Supplemental Figure 3. Nuclei segmentation and RNA segmentation results using wavelet-based segmentation method.

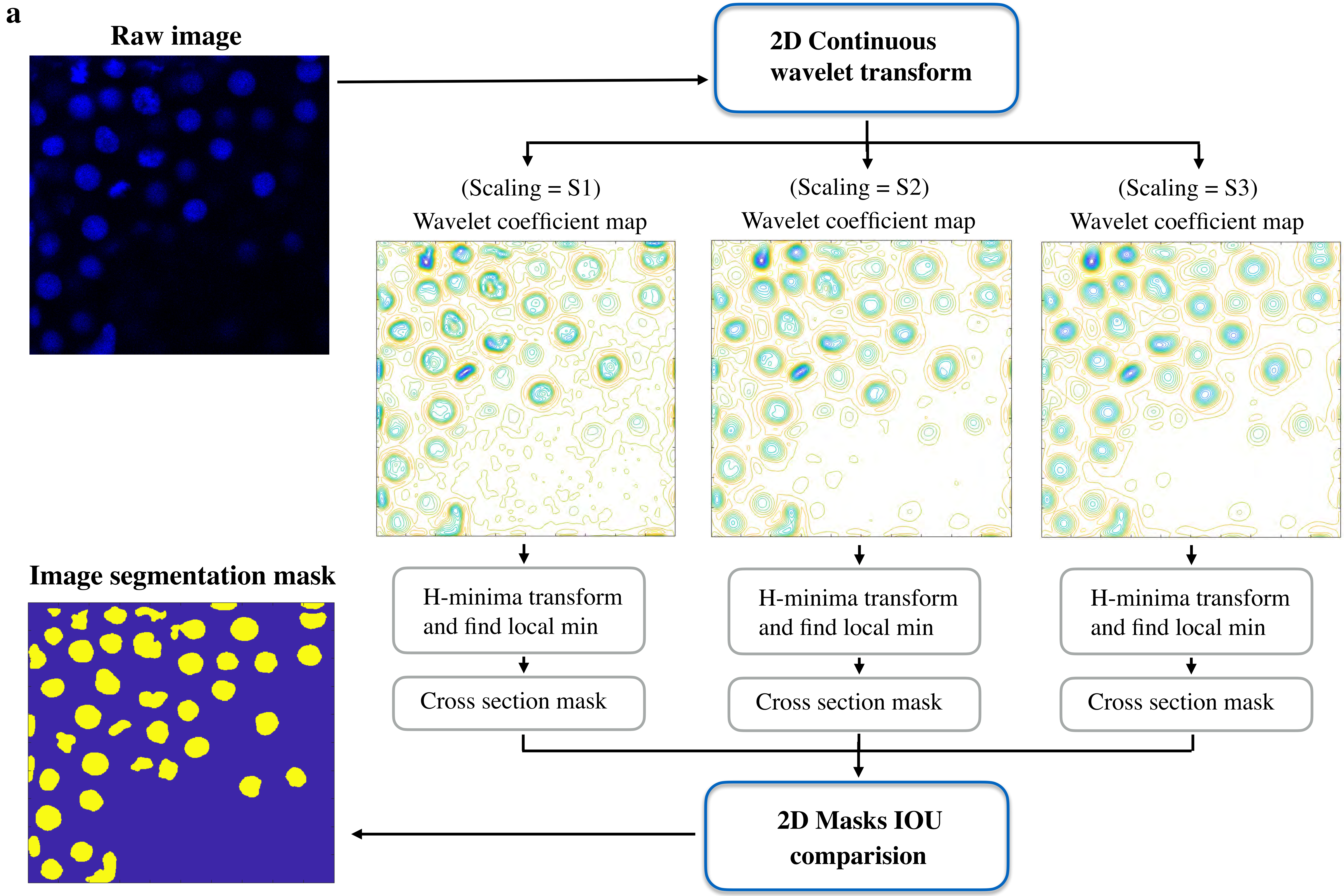

Supplemental Figure 4. Workflow of multi-scale object identification step in wavelet-based segmentation method.

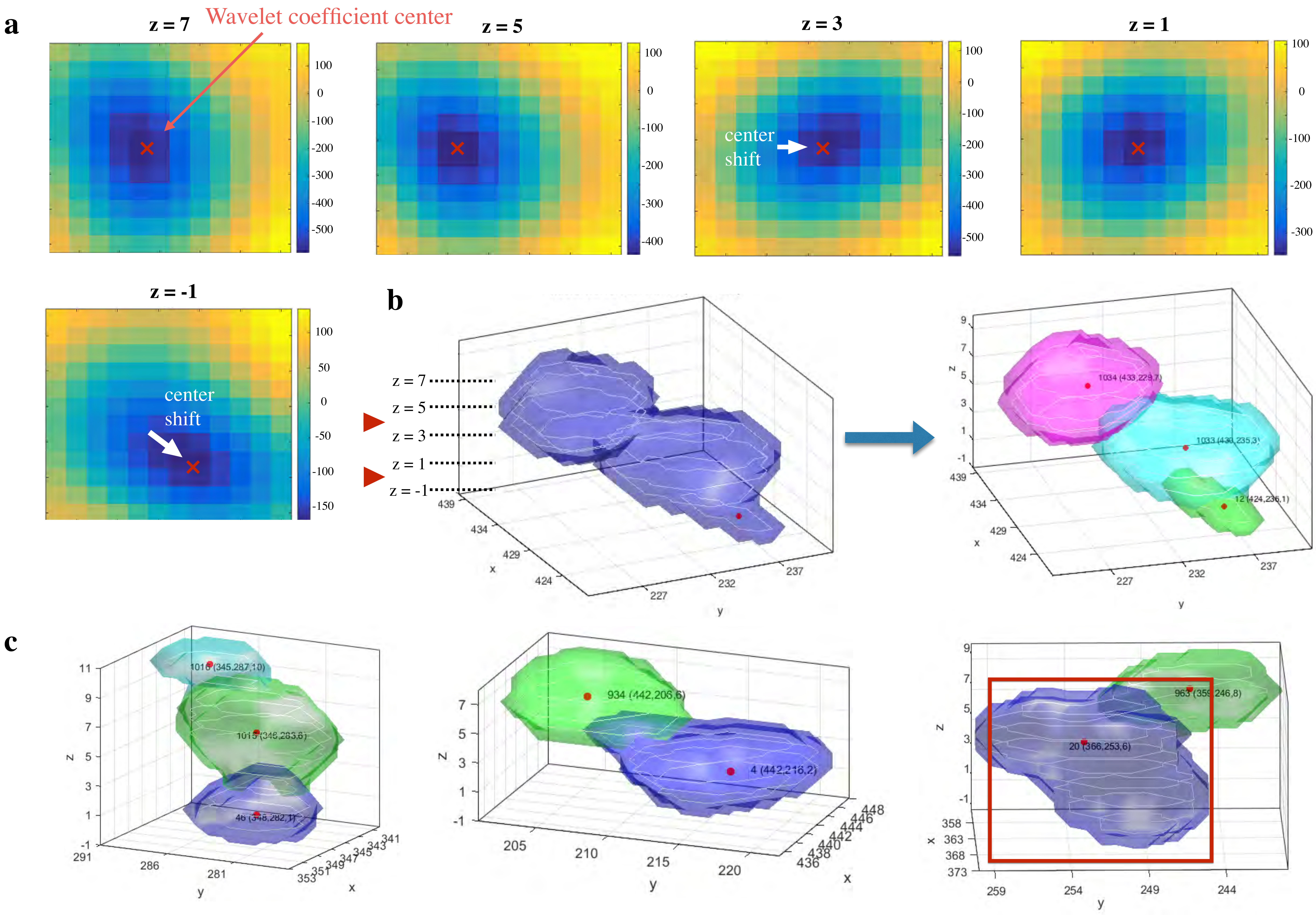

Supplemental Figure 5. First division step based on wavelet coefficient center positions on z-slice of nuclei 3D blob.

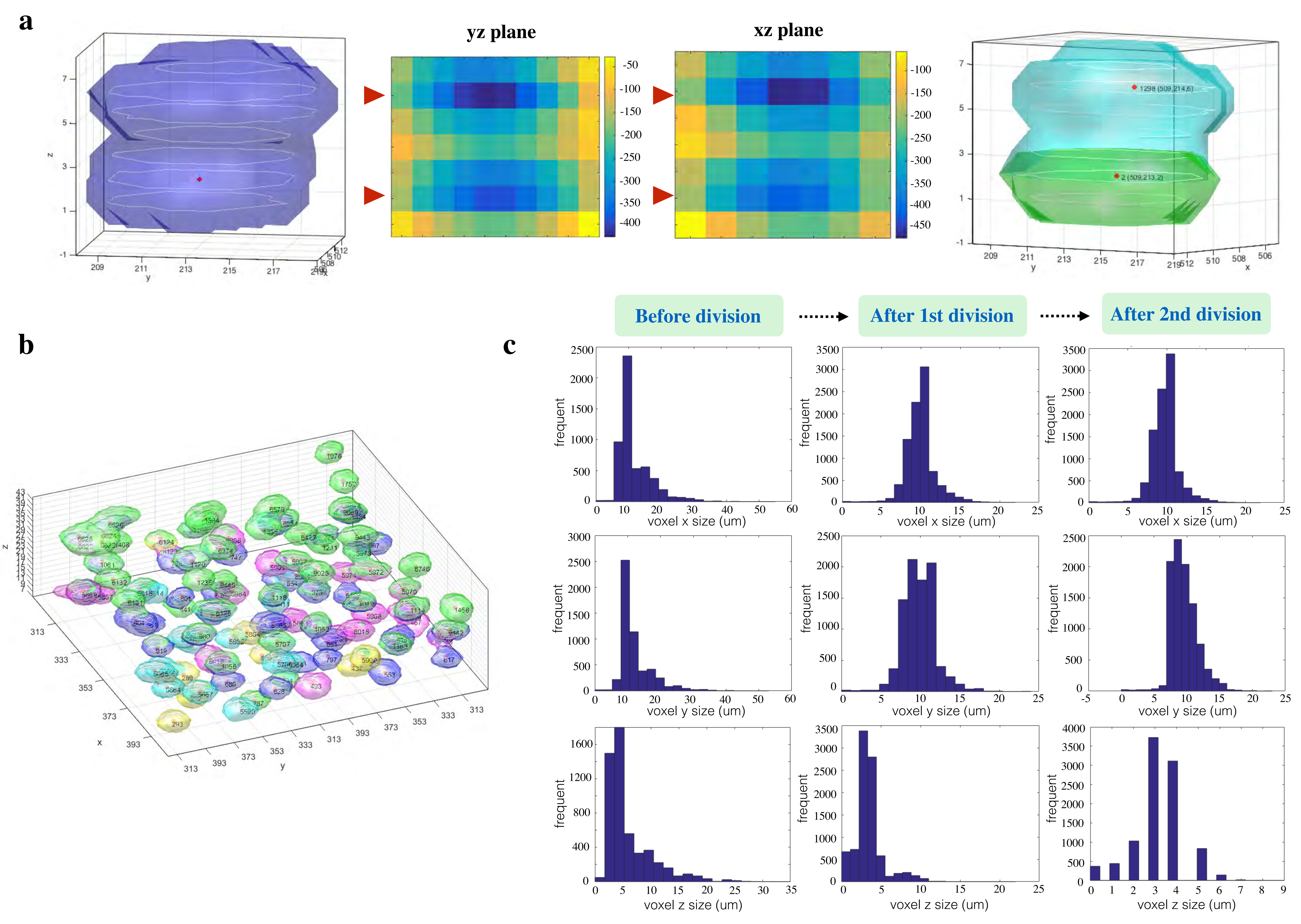

Supplemental Figure 6. Second division step in z direction and the summary of blob size distribution before and after division steps.

a

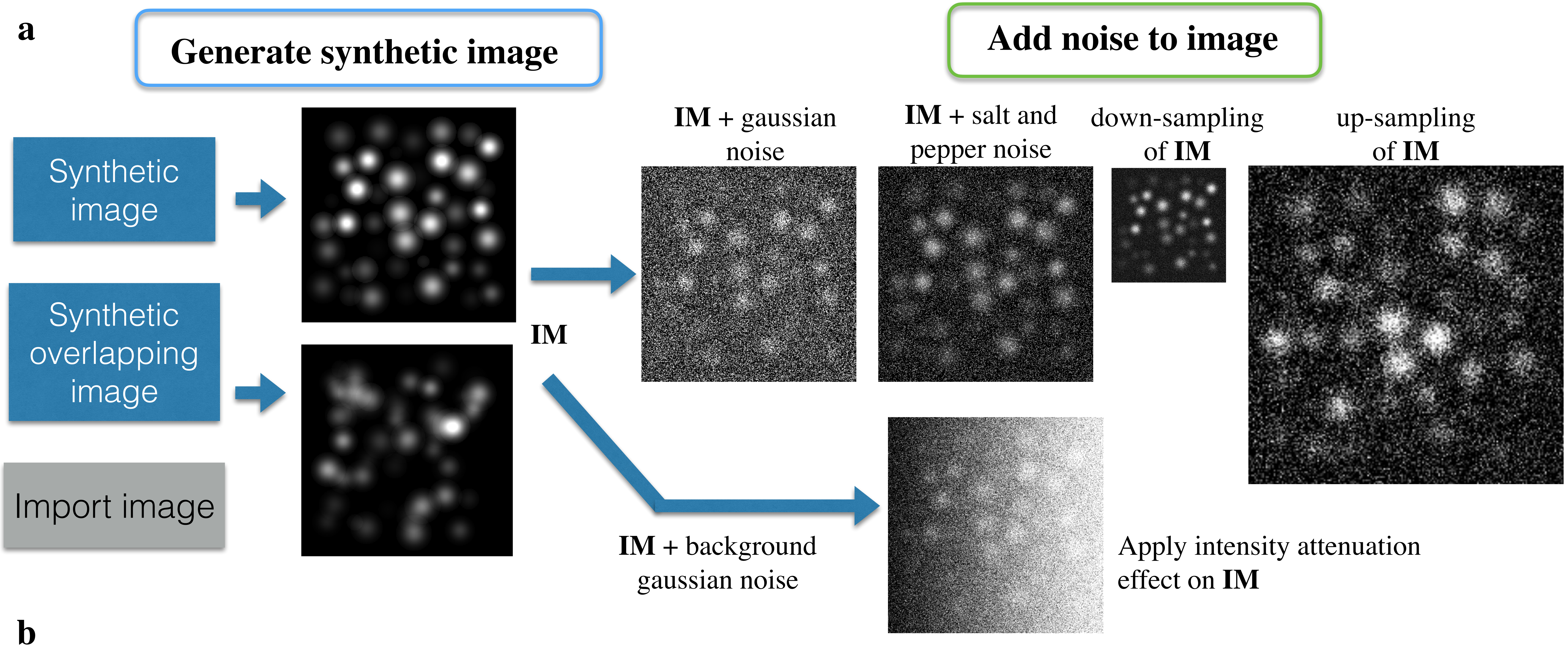

b

Synthetic image property	Synthetic image size: 400x400x40, Image number = 20<br>Nuclei# = 50, Nuclei radius = 30, Nuclei center I = 250<br>Radius randomness = 0.2, Intensity randomness = 0.8
Gaussian noise	Noisy level = 0.01(low), = 0.1(mid), = 0.5 (high)
Salt and pepper noise	Noisy level = 0.01(low), = 0.1(mid), = 0.5 (high)
Optical aberration	Noisy level = 2(low), = 4(mid), = 7 (high)
Nuclei overlap	Nuclei number = 50(low), = 70(mid), = 100 (high)

c

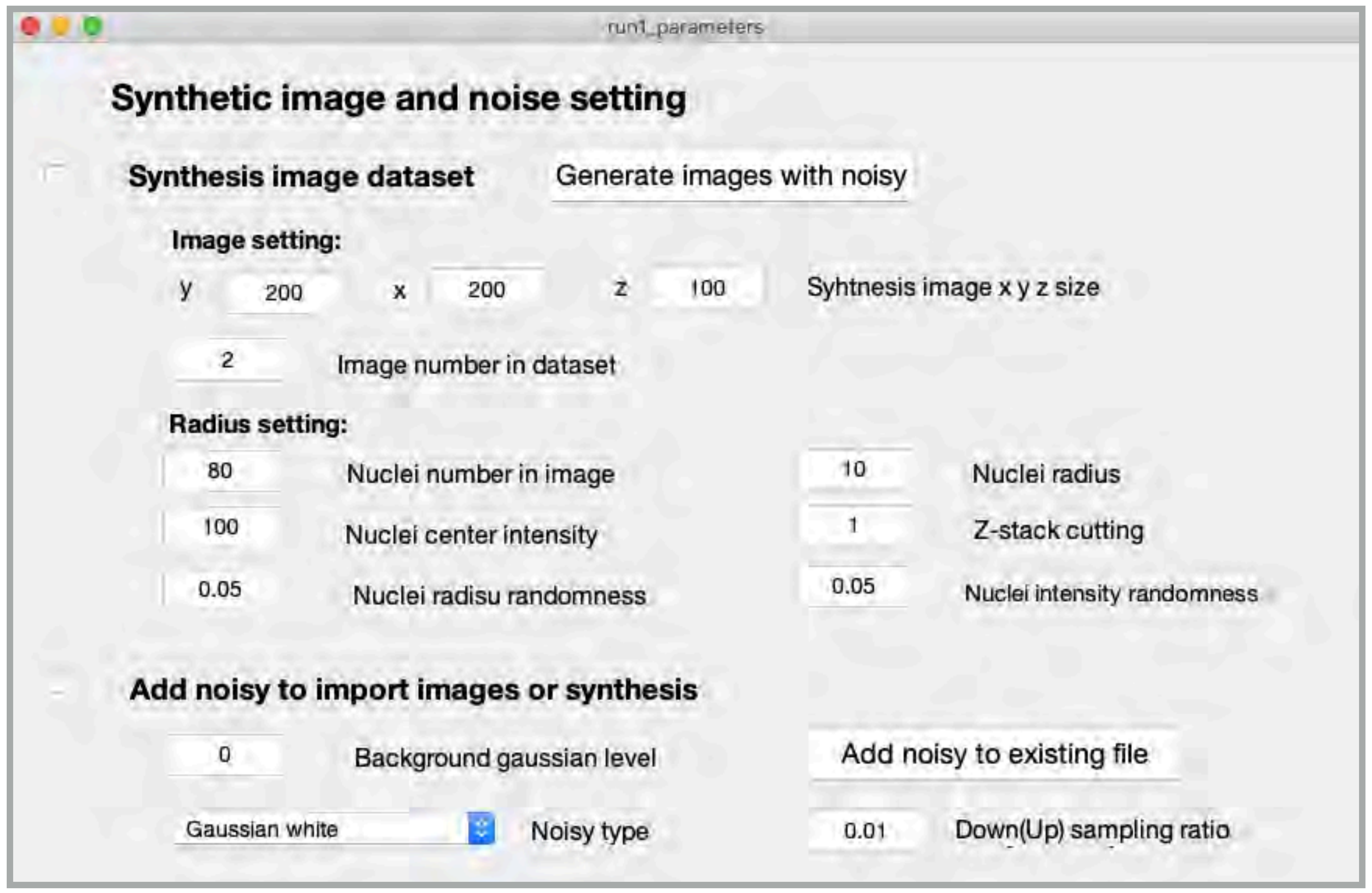

Supplemental Figure 7. The workflow of synthetic data generator and process to add noise in WaveletSEG.

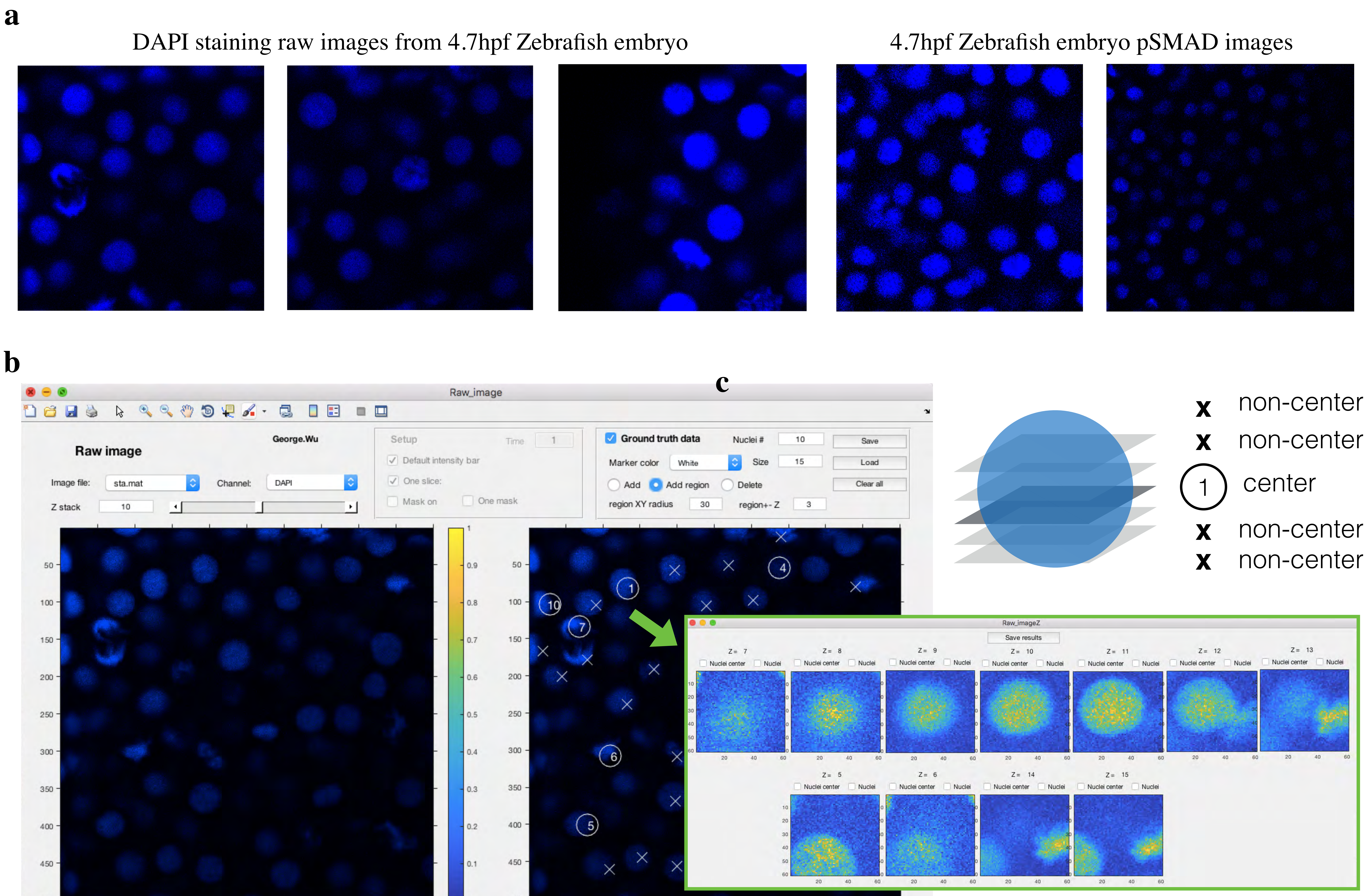

Supplemental Figure 8. Ground truth image dataset we used and the ground truth labeling GUI and 2D segmentation viewer.

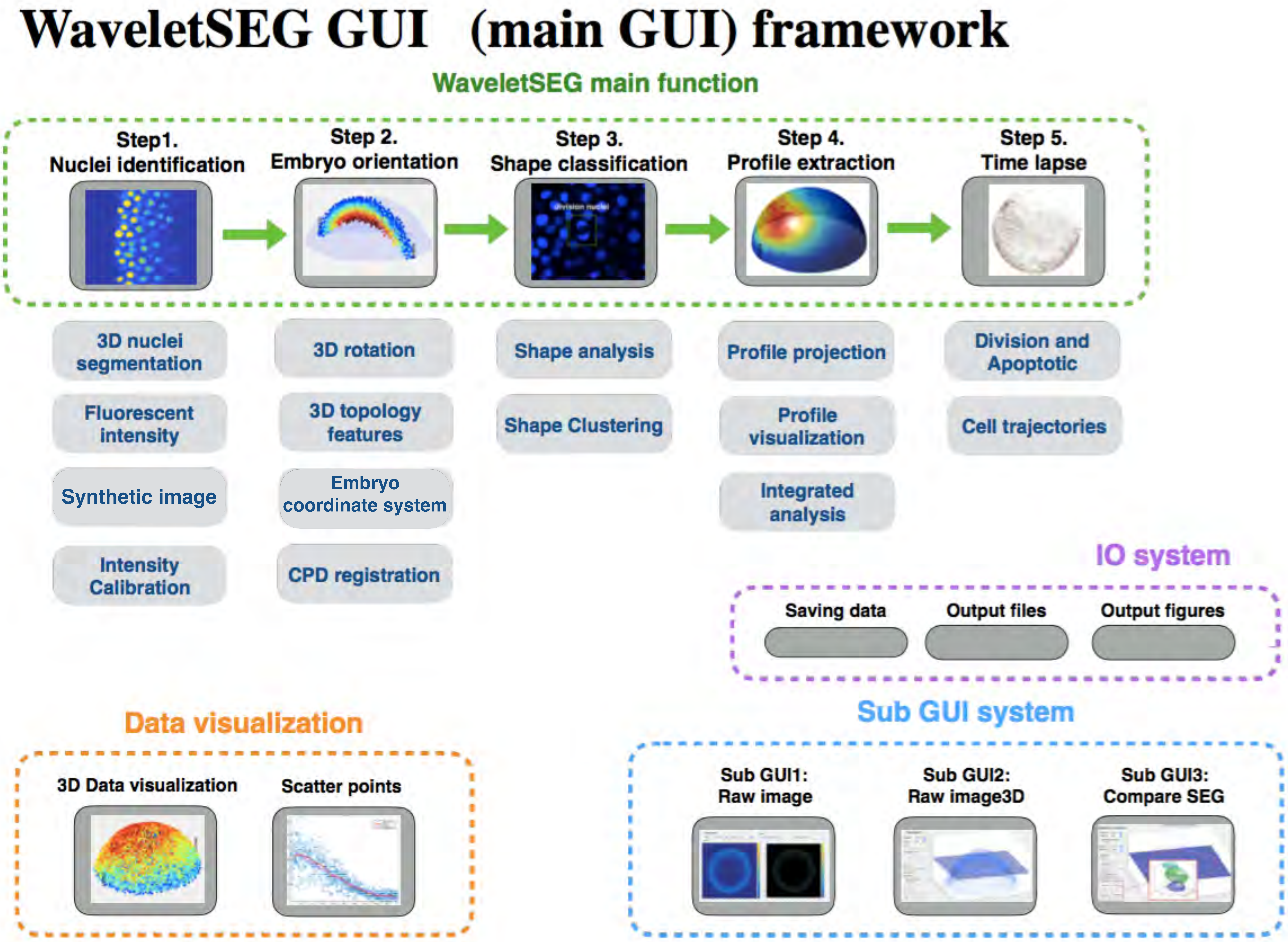

Supplemental Figure 9. WaveletSEG main functions.

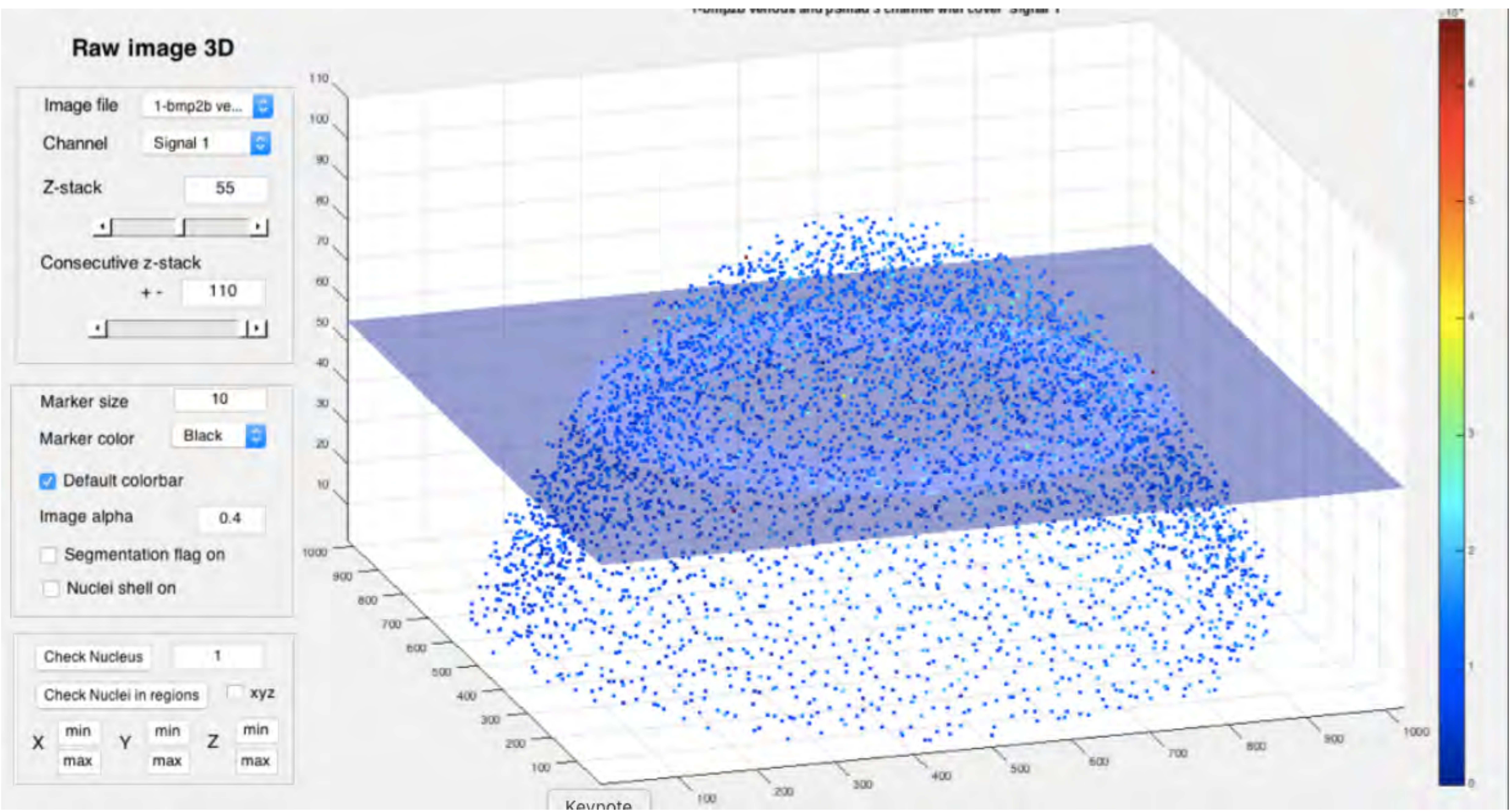

Supplemental Figure 10. Raw image3D sub-GUI.

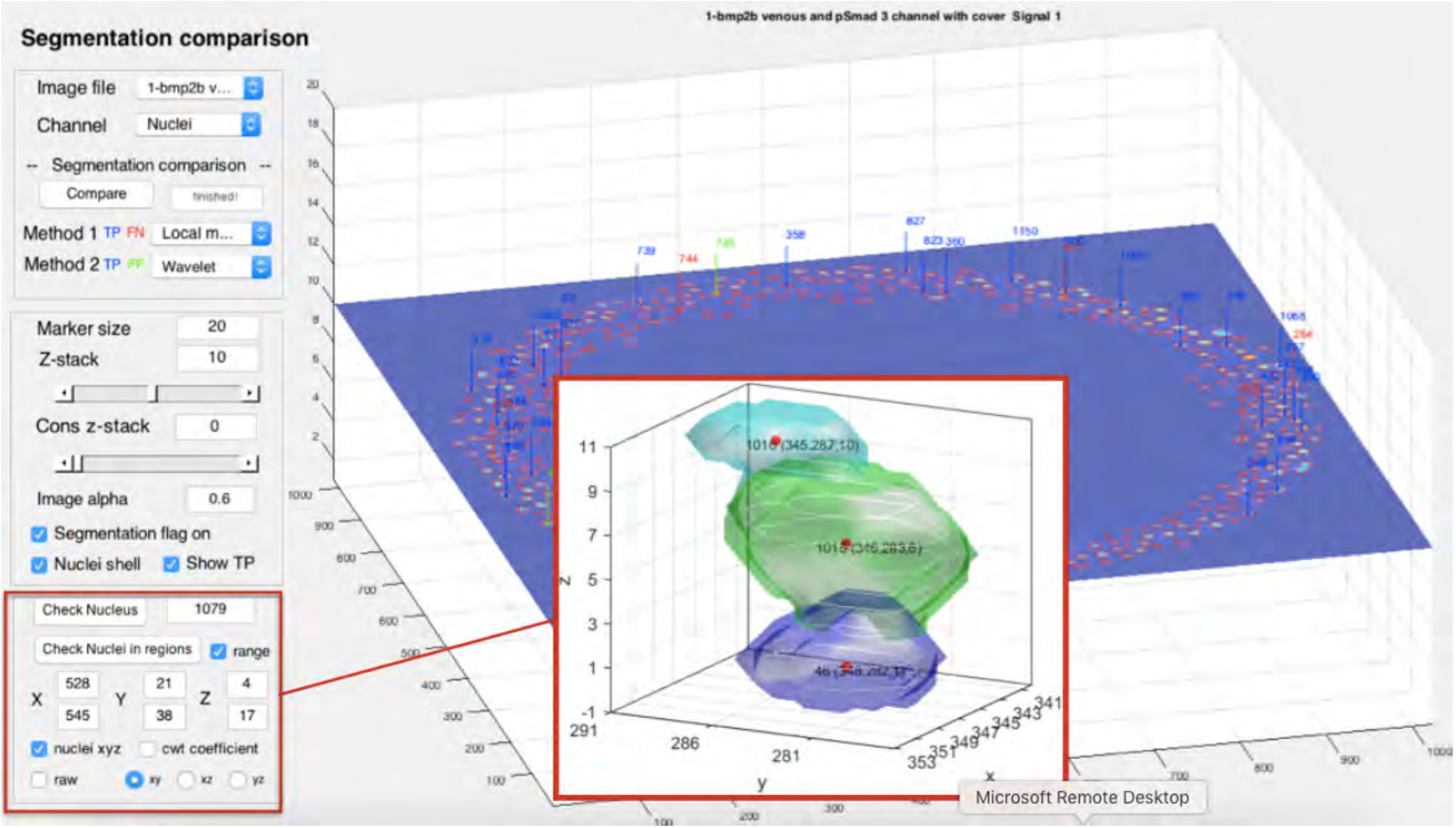

Supplemental Figure 11. Compare SEG sub-GUI.

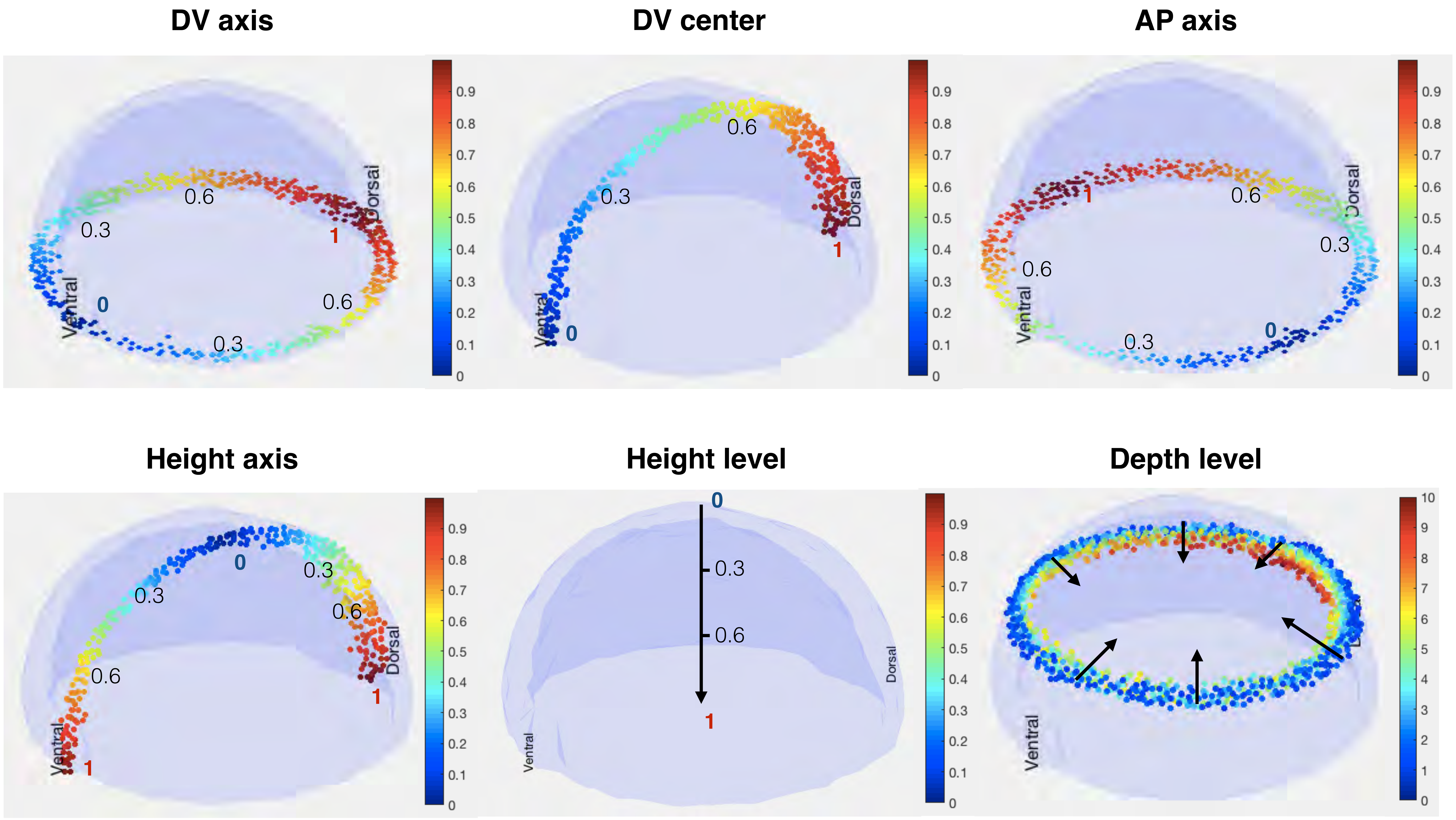

Supplemental Figure 12. Zebrafish embryo coordinate system in WaveletSEG.

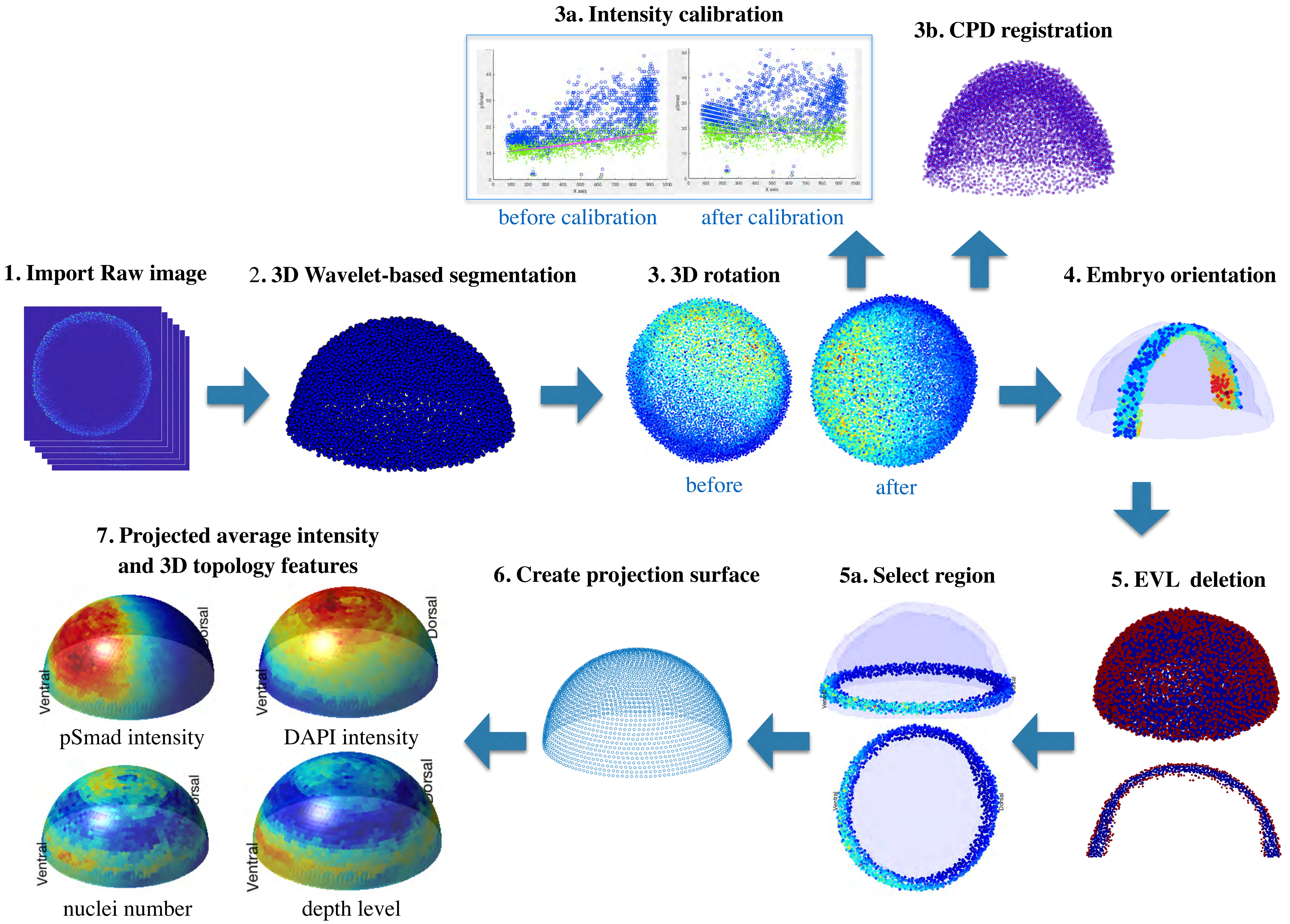

Supplemental Figure 13. Data workflow from 3D whole embryo raw image to projected average intensity distribution in WaveletSEG.

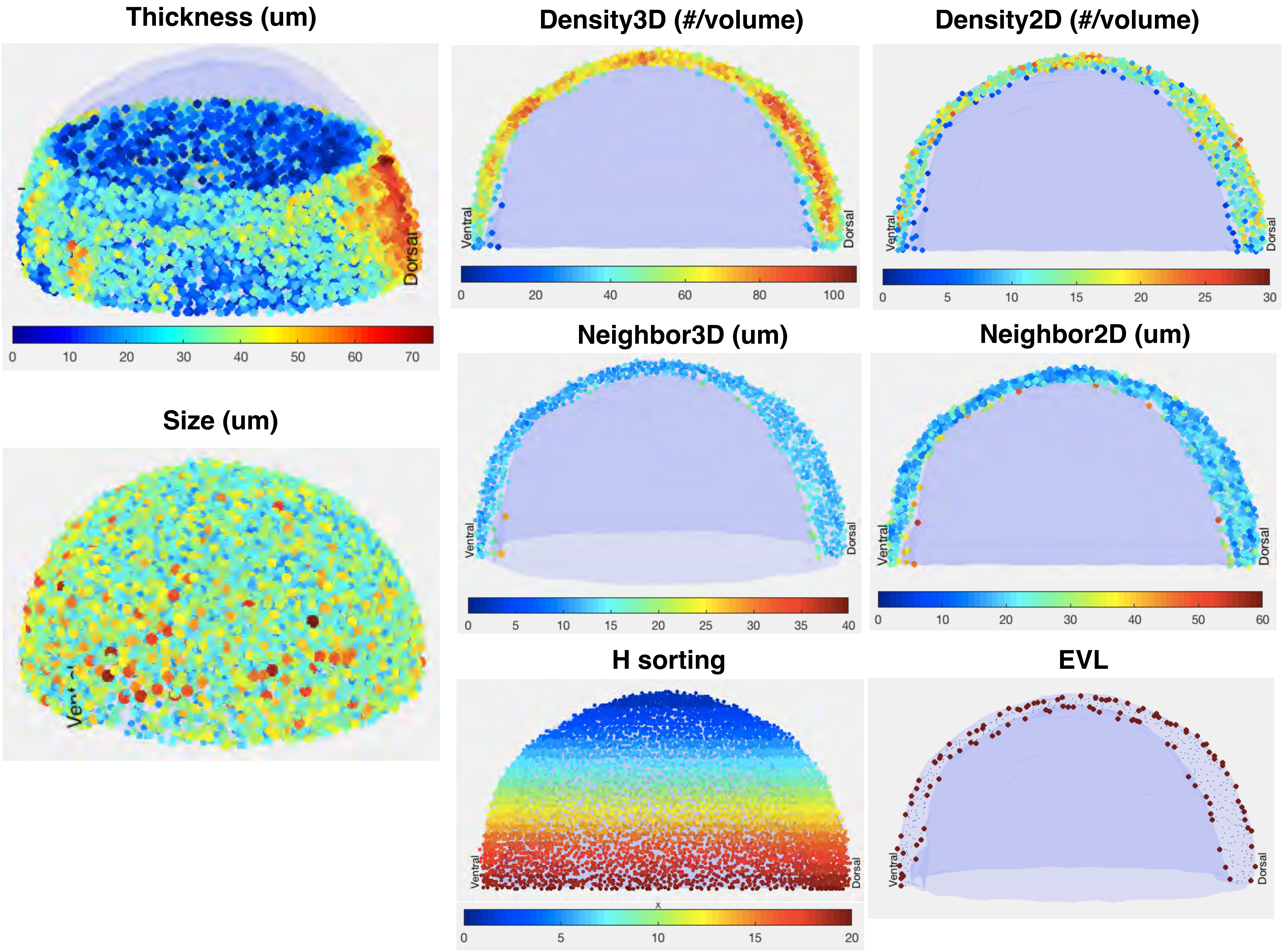

Supplemental Figure 14. 3D topology features used in WaveletSEG.

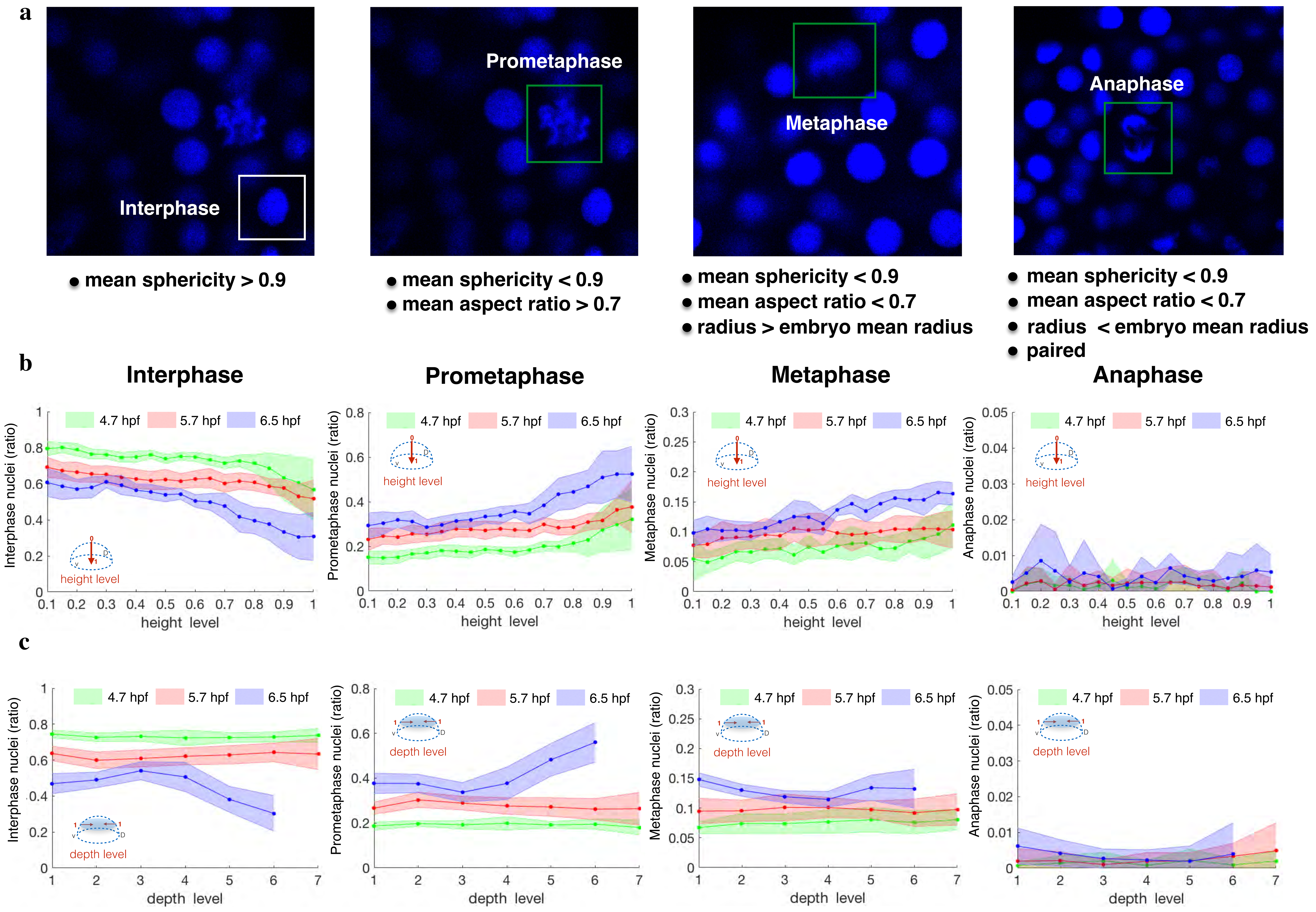

Supplemental Figure 15. Cell cycle phase patterns based on nuclei shape classification results.
