# Supplementary material for: Automatic wavelet-based 3D nuclei segmentation and analysis for multicellular embryo quantification
Source: Sci Rep. 2021 May 10;11:9847. doi: 10.1038/s41598-021-88966-2 (PMC8110989; doi:10.1038/s41598-021-88966-2)

WaveletSEG User Guide

Current version: WaveletSEG v.8
Last updated: February 2020
URL: https://waveletseg.weebly.com/
Support: a3146654@gmail.com

Overview

WaveletSEG is an automatic zebrafish embryo image processing analysis platform that can perform single nuclei to whole embryo scale quantification and topology and shape feature analysis of zebrafish embryos during embryonic development. WaveletSEG is an open-source MATLAB-based imaging research platform and runs on Windows, Mac and Linux systems with complete GUI. Wavelet, Image Processing Toolboxes are required to run WaveletSEG. Here we introduce the step-by-step user guide with example images.

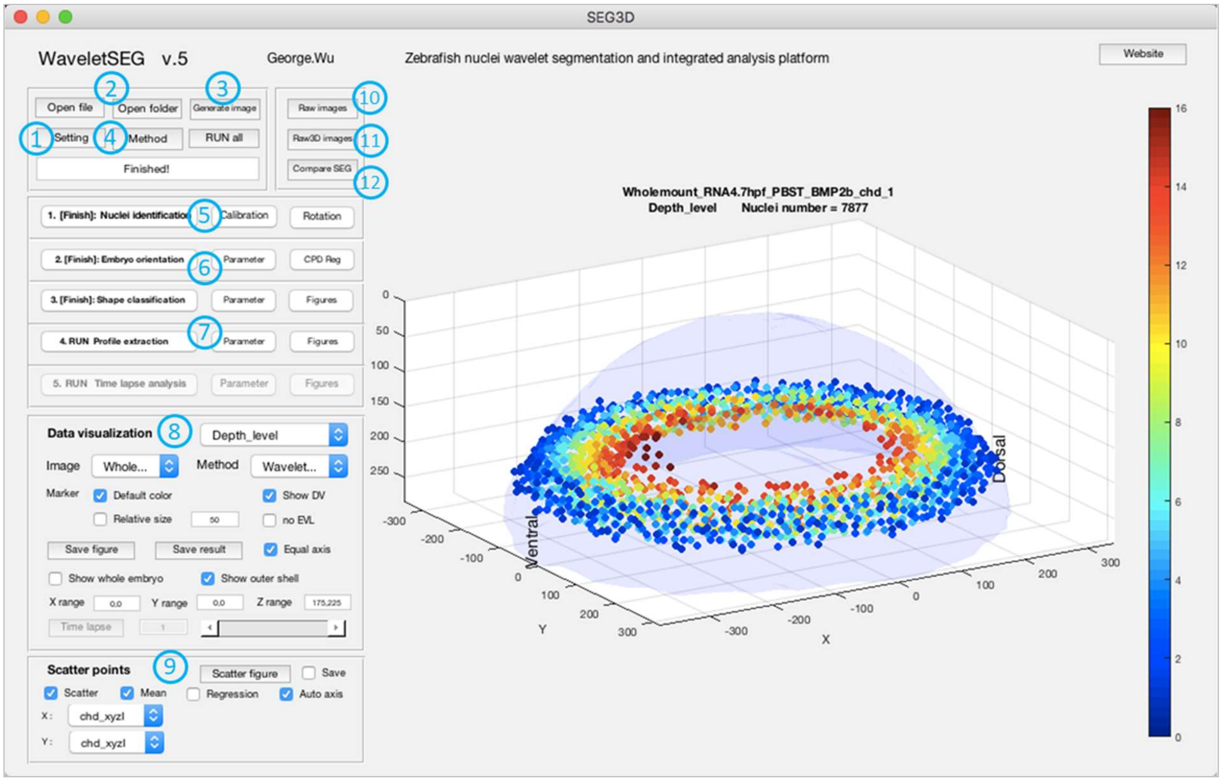

Fig. 1 WaveletSeG software main interface.

0. Example images

Example Image 1,2	tp63_psMad_dapi_8hpf_1.czi, tp63_psMad_dapi_8hpf_2.czi
Channel parameters	Channel number = 3, xy resolution = 2.08, z resolution = 2.2<br>Channel1(Signal 1): tp63<br>Channel2(Signal 2): pSmad<br>Channel3(Nuclei) : DAPI

Example Image 3,4	tp63_psMad_dapi_10hpf_1.czi, tp63_psMad_dapi_10hpf_2.czi
Channel parameters	Channel number = 3, xy resolution = 2.08, z resolution = 2.1<br>Channel1(Signal 1): tp63<br>Channel2(Signal 2): pSmad<br>Channel3(Nuclei) : DAPI

Example Image 5	H2A_GFP_dapi_8gpf_1.czi
Channel parameters	Channel number = 3, xy resolution = 2.08, z resolution = 2.1<br>Channel1(Signal 1): H2A<br>Channel2(Signal 2): GFP<br>Channel3(Nuclei) : DAPI

1. Channel information setting

1. Before importing an image file, click the ‘Setting’ button in the WaveletSEG main interface and input channel information and resolution values in ‘Channel parameter setting’ menu. This step is required before the image import step.
2. Choose the appropriate channel number in the dropdown menu and fill in channel names in empty boxes.
3. Users can import partial parts of an image by setting the two-element vector (min and max values of Z-stack) in ‘Z-stack range’ and the four-element vector(min and max axis limits of x axis, min and max axis limits of y axis) in ‘XY plane range’. Zero value means full range.

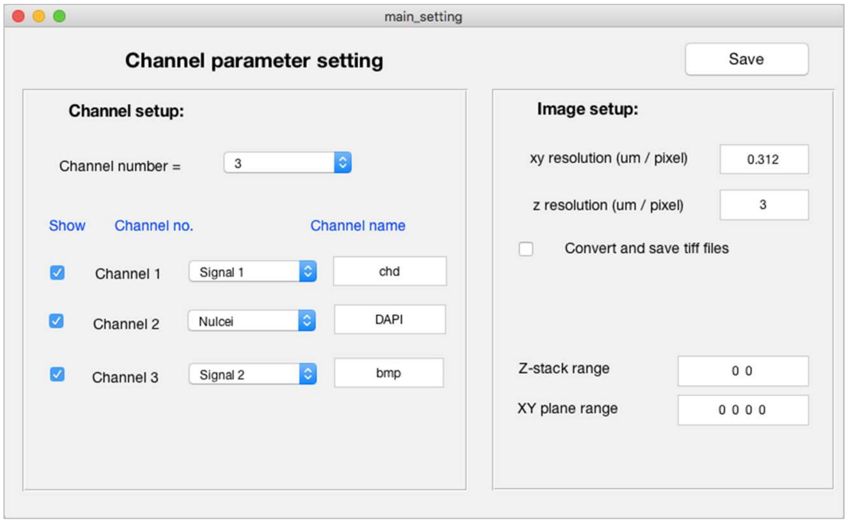

Fig. 2 Channel parameter setting menu.

2. Import file / folder / result

Import image file:

1. Click the 'Open file' button and select single image file with 'tif', 'lsm', or 'czi' image format.
2. New folder will be created to store data and result files after importing image file.
3. 'sta.mat' and 'p.mat' files will be created in this folder.
4. The display box of main GUI will show 'Finished!'.
5. Once loading process is finished, you can check image visualization results or run analysis process.

Import images in folder:

1. Click the 'Open folder' button and select the folder contains 'tif', 'lsm', or 'czi' image files.
2. New folders will be created to store data and result files when importing all image files.
3. 'sta.mat' and 'p.mat' files for every image file will be created in the corresponding folders.
4. The display box of main GUI will show 'Finished!'.
5. Once loading process is finished, you can check image visualization results or run analysis process.

Import result file:

1. Click the 'Open file' button and select 'sta.mat' file in specific folder.
2. All analysis results and data will re-load and the display box of main GUI will show 'Finished!'
3. Analysis process button will show '[Finish]' if you run it and finish it before.
4. Once loading process is finished, you can check image visualization results or run analysis processes.

3. Synthetic image generator for testing and validation

1. Click the ‘Generate image’ button to open ‘Synthesis image and noise setting’ sub-GUI.
2. Users define the synthetic image’s 3D size and number of image and synthetic nuclei signals based on the nuclei number, nuclei radius, and intensity, and the randomness or variability of nuclei radii and intensity between 0 to 1.
3. 3D Gaussian function with nuclei intensity and standard deviation equal to nuclei radius r × 0.4 is added to the synthetic image.
4. Users can add both white noise and user-defined noise types with user-defined noise level noise density (ND) onto the synthetic image.

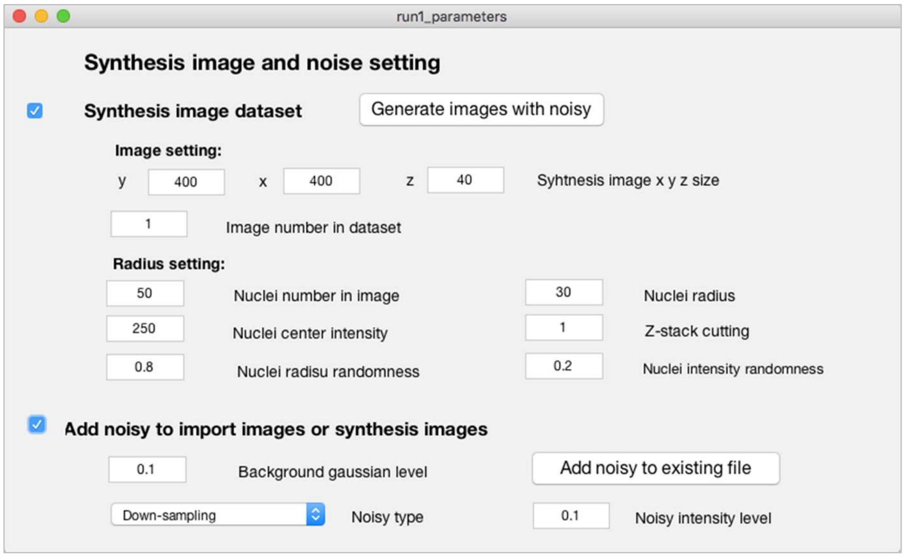

Fig. 3 Synthetic image and noise setting sub-GUI.

4. Select Segmentation method

1. Before running the segmentation step, click ‘Method’ button in WaveletSEG and to open the ‘Segmentation method setting’ menu. This step is required before the ‘Nuclei identification’ step.
2. Select the segmentation methods by clicking the checkboxs before the four methods including Point-wise method, wavelet method, Threshold(Otsu) method, and DS method. Multiple selections accepted.
3. Input the parameter values for segmentation method you selected.
4. Click the ‘X’ and leave this menu.

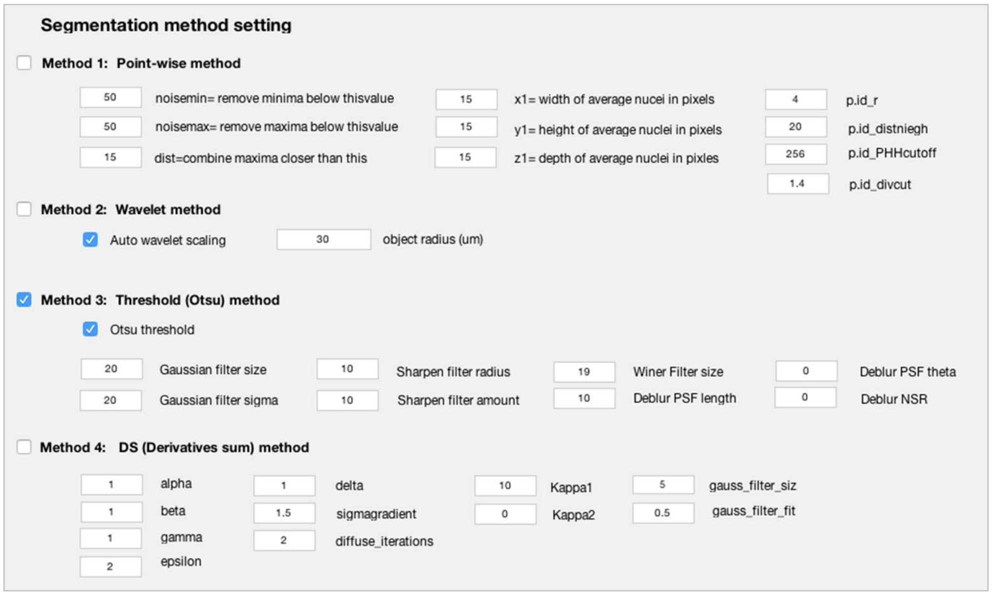

Fig. 4 Segmentation method setting menu.

5. Embryo axis 3D rotation

1. Click the ‘Rotation’ button in WaveletSEG to open ‘Embryo 3D rotation’ menu.
2. Input the clockwise rotation angle (unit: degree) about the axis. Example: Input ‘30’ in Z axis clockwise rotation degree means clockwise rotate 30 degree on XY plane.
3. Click the ‘Rotate’ button in ‘Embryo 3D rotation’ menu to rotate the nuclei 3D points and close the menu.
4. Click ‘Set default’ button to recovery nuclei 3D point positions into un-rotated positions.
5. Please re-run ‘2. RUN Embryo orientation’ step after the rotation step.

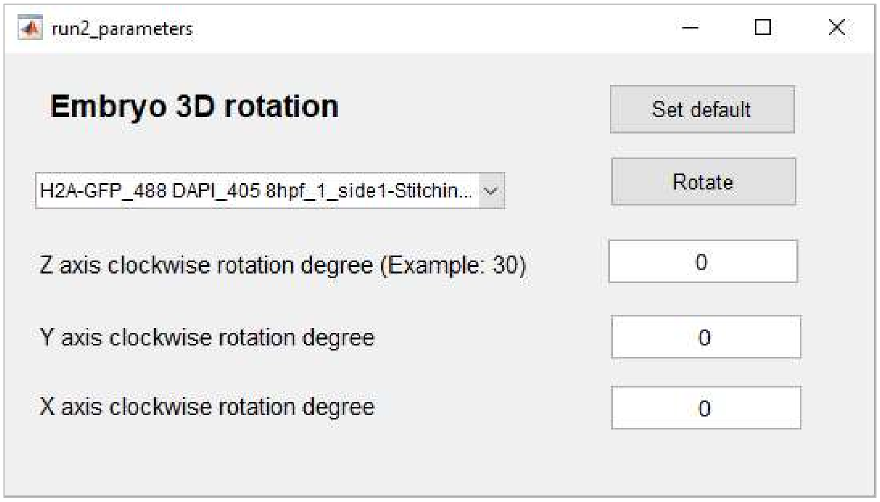

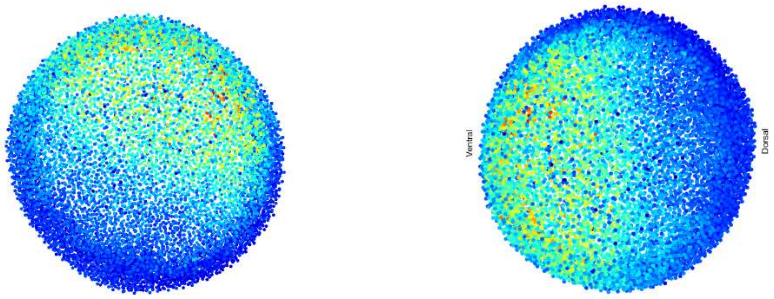

Fig. 5 Embryo 3D rotation menu.

6. Embryo orientation setting

1. Click the ‘Parameter’ button behind ‘2. RUN Embryo orientation’ button in WaveletSEG to open the ‘RUN2 parameters setting’ menu.
2. Input / Change the parameter values in 3D topology features and CPD registration and push ‘Update parameters’ button to update new parameter values. Users can also recover parameter values as default values by clicking ‘Restore default’ button.
3. Users can delete segmented nuclei by inputting nuclei IDs or Z slice range and click ‘Update parameters’ button.
4. Users can recover last time deleted nuclei by clicking ‘Recover delete’ button.
5. Click the ‘X’ and leave this menu.

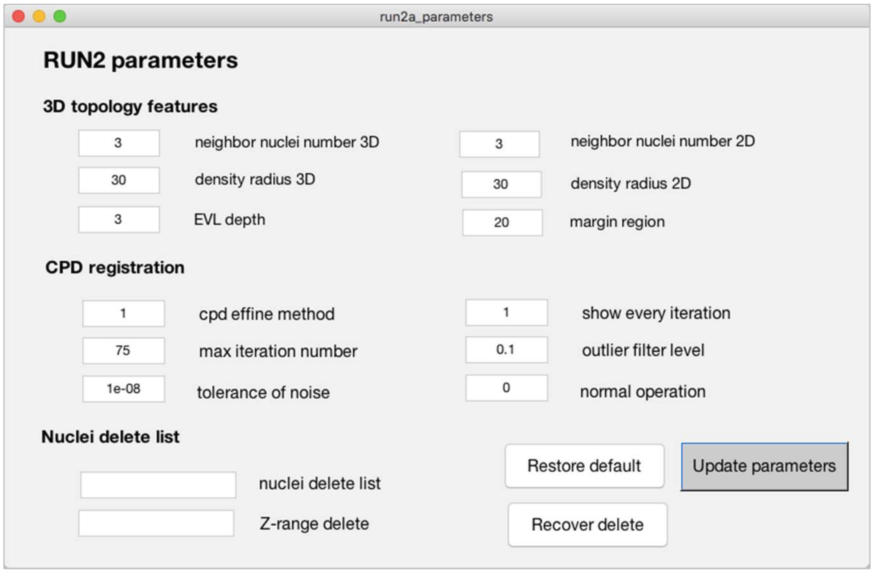

Fig. 6 RUN2 parameters setting menu.

7. Nuclei shape classification setting

1. Click the ‘Parameter’ button behind ‘3. RUN Shape classification’ button in WaveletSEG to open the ‘RUN3 parameters’ menu.
2. Input / Change the parameter values in nuclei shape classification threshold and SOM cluster setting and push ‘Update parameters’ button to update new parameter values. Users can also recover parameter values as default values by clicking ‘Restore default’ button.

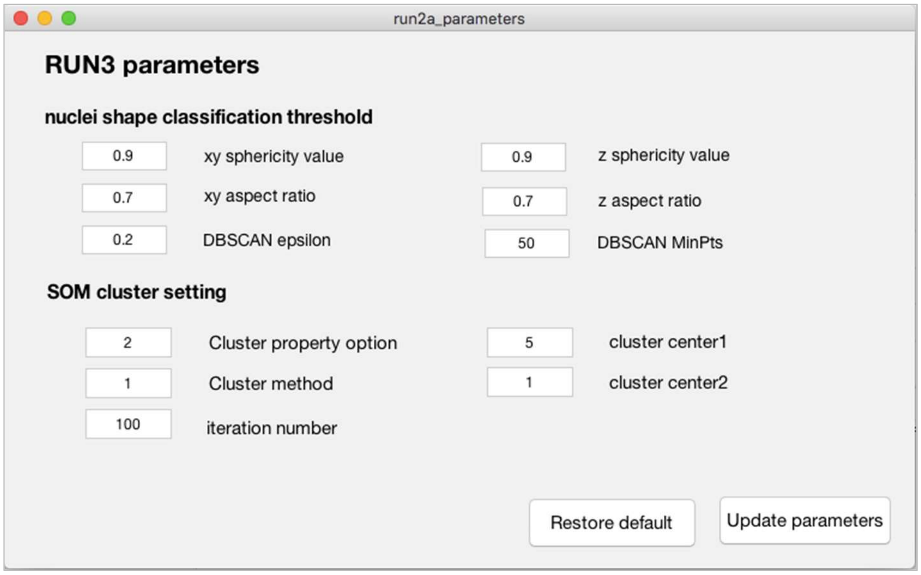

Fig. 7 RUN3 parameters setting menu.

8. Data visualization

1. The right-side window of WaveletSEG is the main data visualization window, where segmented nuclei positions are displayed as a 3D point cloud.
2. In the left middle side of WaveletSEG main interface is the data visualization panel.
3. Select specific channel and the segmentation method used from the dropdown menus.
4. Choice the channel, embryo orientation or shape classification feature to display in The ‘Data visualization’ dropdown menu.
5. Click ‘Show DV’ checkbox to mark the ventral and dorsal positions on data visualization window.
6. Click the ‘no EVL’ checkbox to remove the EVL layer nuclei on data visualization window.
7. Unclick the ‘Show whole embryo’ checkbox to display sub-regions of the whole embryo.
8. Click ‘Save figure’ to save display figure. Click ‘Save result’ to save result in mat files.

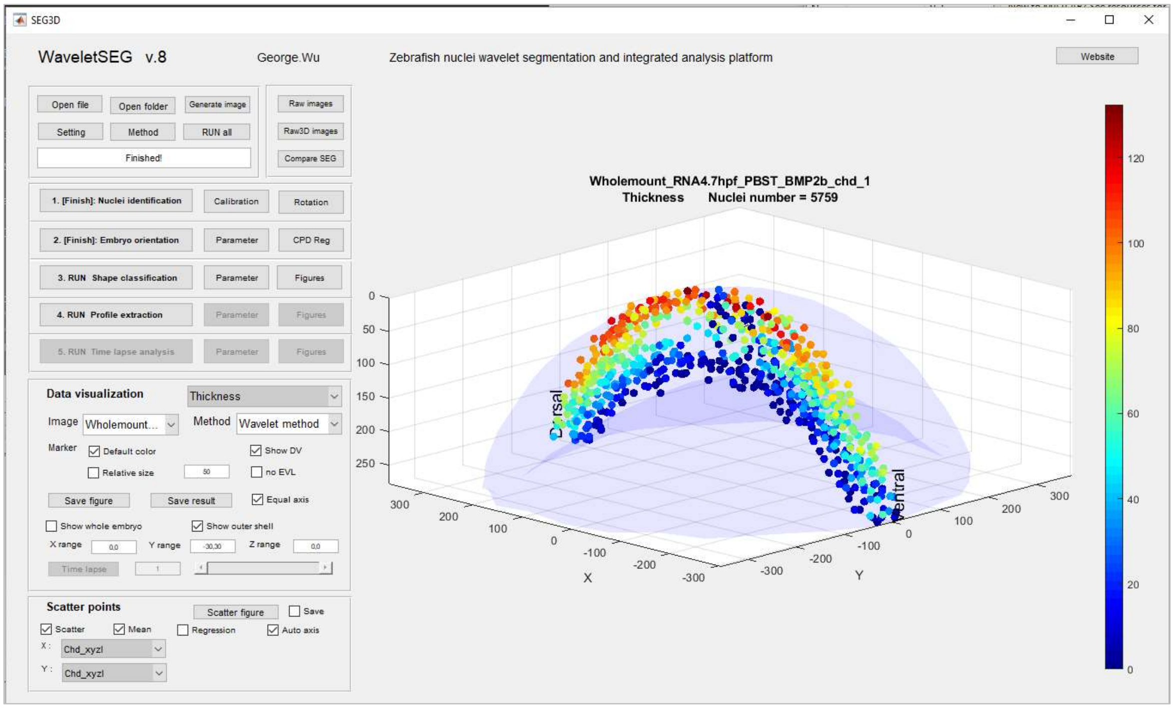

Fig. 8 Data visualization panel – Thickness spatial distribution.

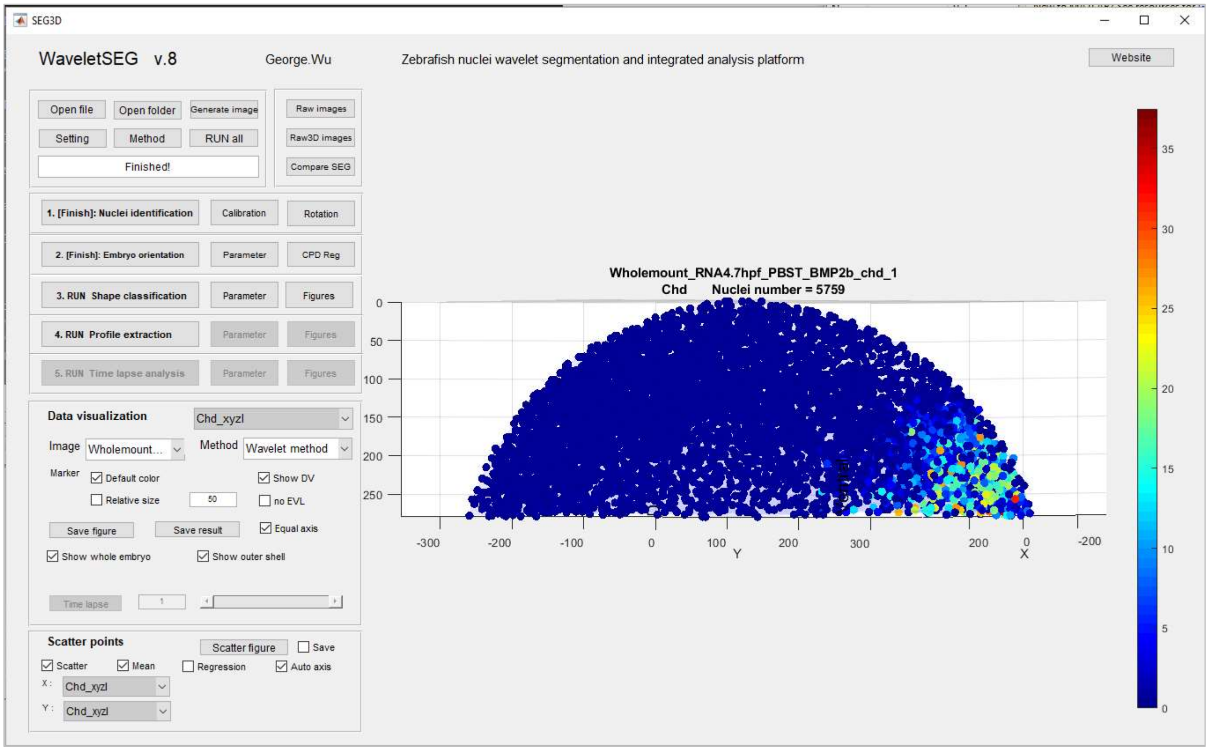

Fig. 9 Data visualization panel – Chd intensity spatial distribution.

9. Scatter point generator

1. In the left bottom side of WaveletSEG main interface is the scatter point generator panel.
2. Select the scatter point x-axis feature in the first dropdown menu.
3. Select the scatter point y-axis feature in the second dropdown menu.
4. Click ‘Mean’ or ‘Regression’ checkboxes to add the mean value line or the regression line.
5. Click the ‘Scatter figure’ to generate scatter point figure.

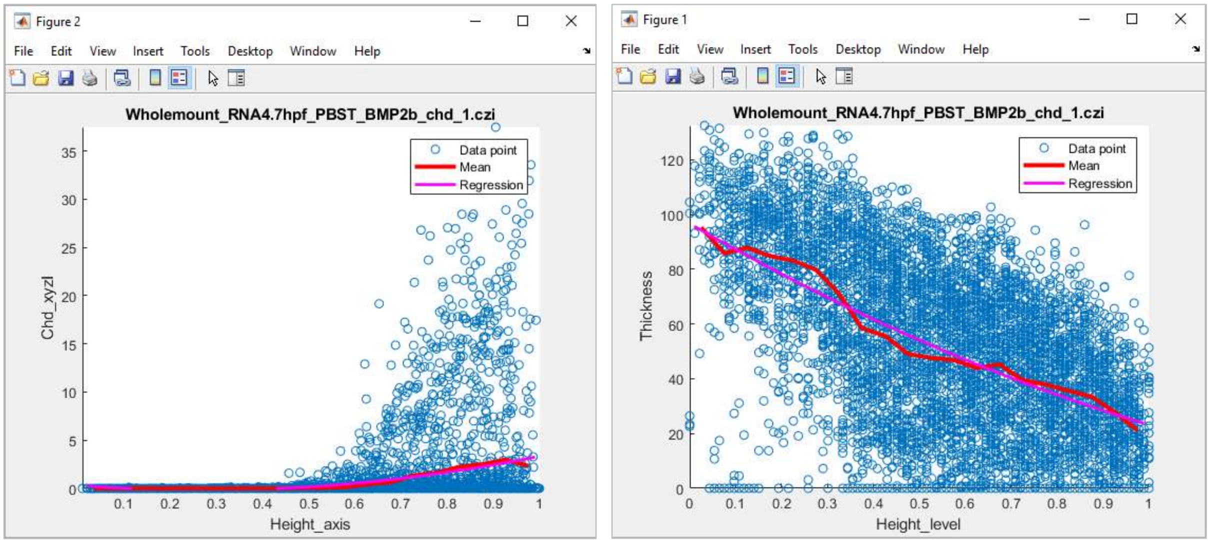

Fig. 10 Scatter points examples.

10. Ground truth labeling GUI and 2D segmentation viewer

2D segmentation viewer:

1. Click the button ‘Raw images’ in WaveletSEG main interface.
2. Select specific channel and the segmentation method used from the dropdown menus.
3. Left window of ‘Raw image’ sub-GUI shows the raw image, and the right window shows both raw image and segmentation labels and masks.
4. Click the ‘Default intensity bar’ checkbox to modify the windows intensity range.
5. Click the ‘Mask on’ checkbox to visualize segmentation masks, or click the ‘One mask’ to visualize individual segmentation mask.

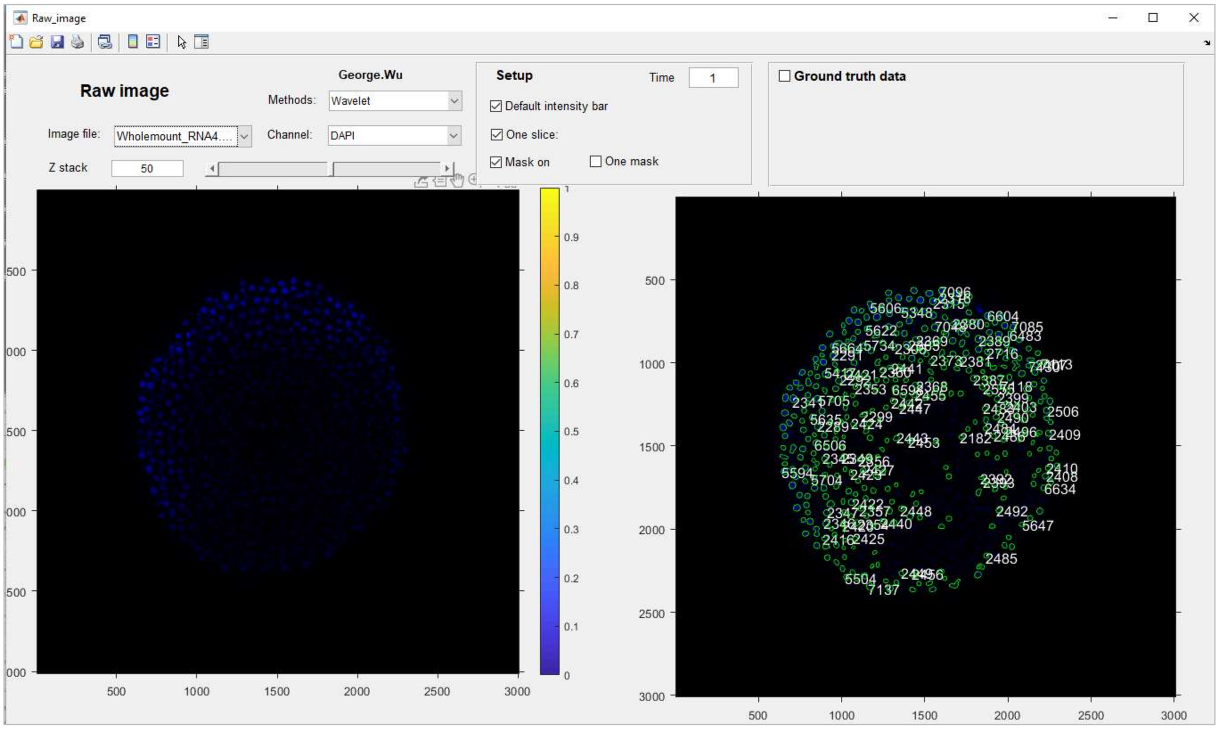

Fig. 11 Raw images sub-GUI as 2D segmentation viewer.

Ground truth labeling tool:

1. Click the button ‘Raw images’ in WaveletSEG main interface.
2. Select specific channel and the segmentation method used from the dropdown menus.
3. Click the ‘Ground truth data’ checkbox to open the ground truth labeling mode.
4. Both left and right windows of ‘Raw image’ sub-GUI shows the raw image.
5. When selecting ‘Add’ mode, users can mutually label nuclei center by clicking on the right window. There are two main types of labeling: nuclei center labeling, which shows the nuclei index, and non-center nuclei labeling, which is marked as a cross symbol.
6. When selecting ‘Add region’ mode, one sub graphical user interface (GUI) will pop up in a specific region with a range of z-stack planes after clicking on the right window. Users can directly assign the labeling by clicking.
7. Users can save ground truth labeling works by clicking ‘Save’ button and load saved results by clicking ‘Load’ button.

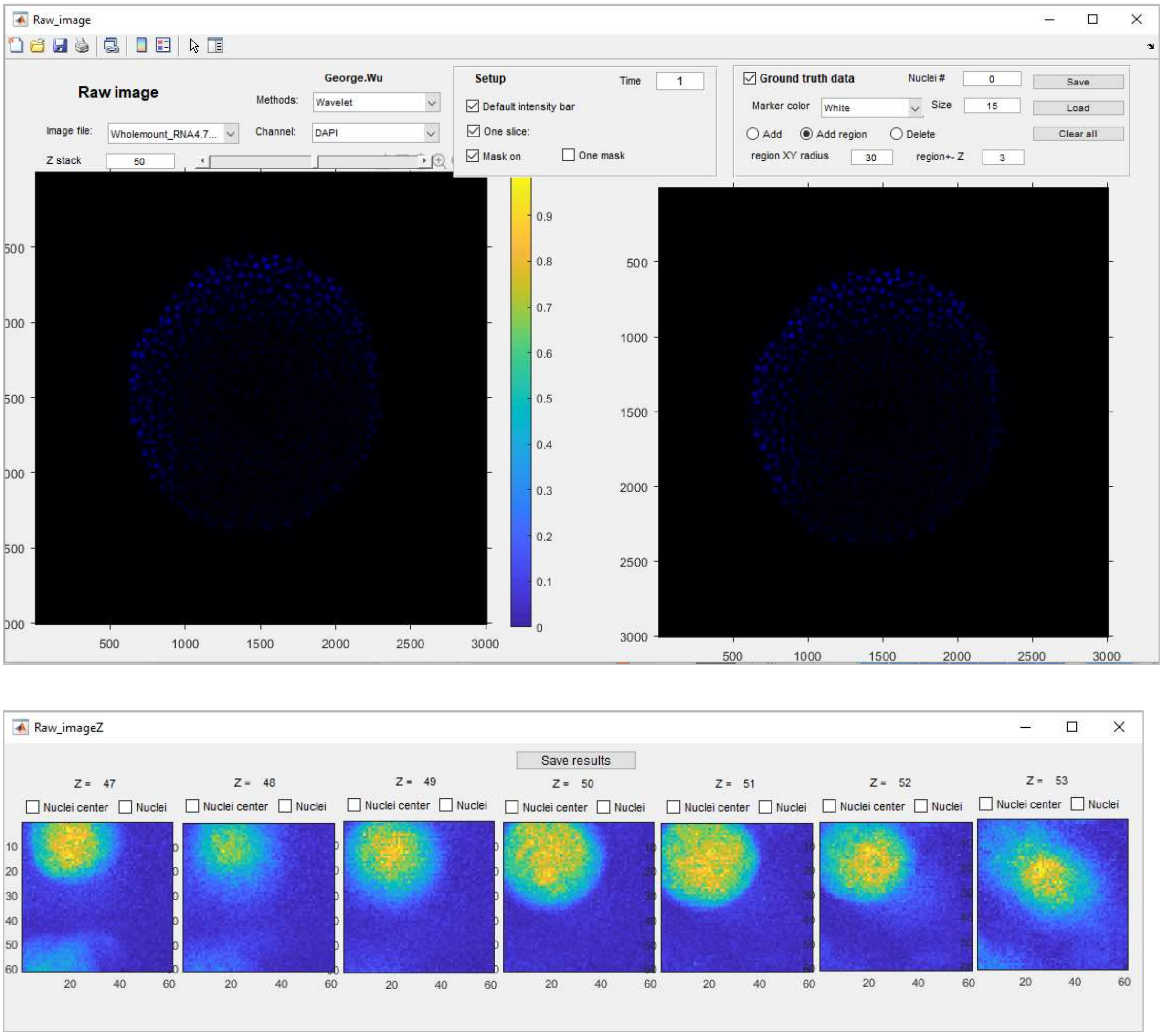

Fig. 12 Raw images sub-GUI as Ground truth labeling tool.

11. Raw image3D sub-GUI

1. Click the button “Raw3D images” in WaveletSEG main interface.
2. The right side window of ‘Raw image3D’ sub-GUI is the main data visualization window, where segmented nuclei positions are displayed as a 3D point cloud.
3. The horizontal plane is a raw image z-slice that enables the users to validate and compare 3D segmented nuclei with original raw image z-slices.
4. Left side of ‘Raw image3D’ sub-GUI is the main control panel which can choice different marker properties and visualize segmentation masks.
5. Button left of the sub-GUI is the 3D surface rendering tool panel.

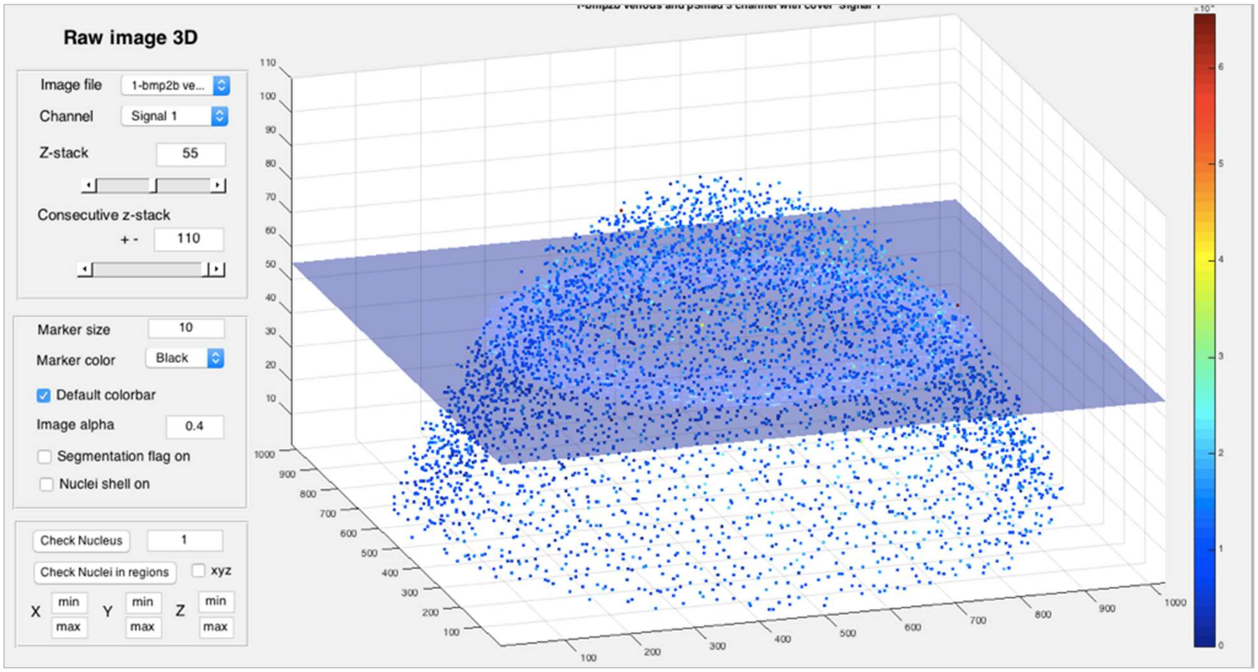

Fig. 13 Raw image3D sub-GUI.

12. Compare SEG sub-GUI

1. Click the button "Compare SEG" in WaveletSEG main interface.
2. On the left side of segmentation comparison is the main control panel with Method 1 and Method 2 dropdown menus to compare their segmentation results.
3. The right-side window of 'segmentation comparison' sub-GUI is the main data visualization window, where segmented nuclei positions are displayed as a 3D point cloud.
4. When comparing two segmentation methods, segmented nuclei will be marked as blue (True Positive), red (False Negative), or green (False Positive). TP rate and precision will automatically be saved in data files.
5. Button to the left of the sub-GUI is the 3D surface rendering tool panel. 3D surface rendering of each segmented nuclei can be conducted by selecting a nuclei spot number or the xyz axis range after 3D reconstruction in this control panel.

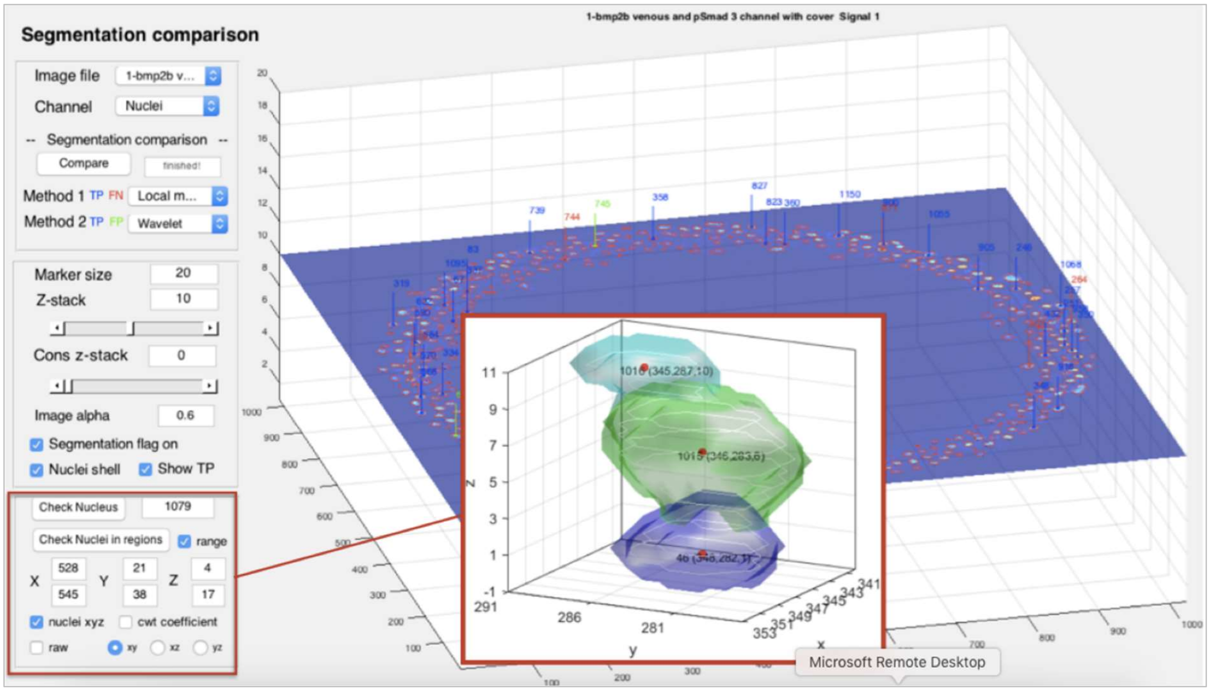

Fig. 14 Compare SEG sub-GUI.

13. Matlab settings for Windows system

Open up matlab and click on setpath on the top.

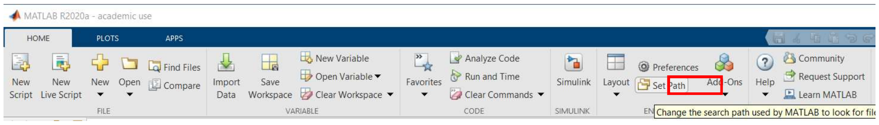

Click on Add with Subfolders, select WaveletSEG folder. Then press save to save the path.

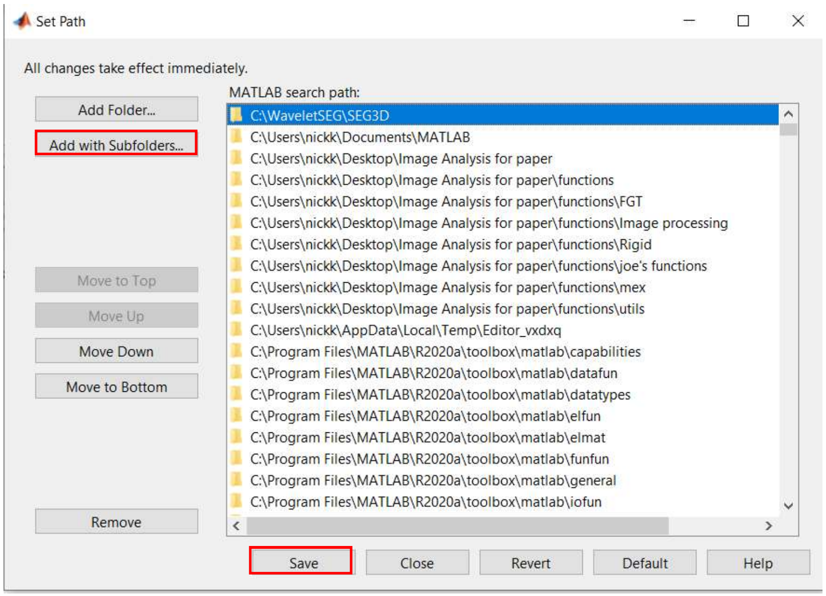

Supplement: Supplementary file 2 — Supplementary Information 2. [file 41598_2021_88966_MOESM2_ESM.pdf]
